# Global prevalence of *Salmonella* spp. and *Escherichia coli* in reptile taxa: a systematic review and meta-analysis

**DOI:** 10.3389/fmicb.2026.1940090

**Published:** 2026-10-28

**Authors:** Lungile N. Mlangeni, Cormac Price, Jane Nkhebenyane, Tsepo Ramatla, Che Weldon

**Affiliations:** 1 Unit for Environmental Sciences and Management, North-West University, Potchefstroom, South Africa; 2 School of Biological and Environmental Sciences, University College Dublin, Belfield, Dublin, Ireland; 3 Centre for Applied Food Safety and Biotechnology, Department of Life Sciences, Central University of Technology, Bloemfontein, South Africa

**Keywords:** *Escherichia coli*, global prevalence, meta-analysis, reptiles, *Salmonella* spp., zoonotic reservoirs

## Abstract

**Background:**

Reservoirs of zoonotic agents have been well-described in reptiles; however, global consensus across reptile taxa is lacking. In this systematic review and meta-analysis, the global prevalence of Salmonella spp. and *Escherichia coli* were estimated in reptiles to provide an overview of their occurrence and potential public health relevance.

**Methods:**

The Preferred Reporting Items for Systematic Reviews and Meta-Analyses (PRISMA) guidelines were adhered to. Five online databases PubMed, Scopus, ScienceDirect, Google Scholar, and Medline were thoroughly searched to conduct meta-analyses. Data on prevalence were pooled using the random-effects model and heterogeneity was quantified using *I^2^* statistics to ensure robustness of the data. Further, the publication bias was carefully assessed by Egger’s linear regression and Begg’s rank correlation tests.

**Results:**

The pooled prevalence of *Salmonella* spp. and *Escherichia coli* was examined using Comprehensive Meta-Analyses software v4.0. A total of 189 studies met the criteria for inclusion in this meta-analysis. Across these studies, a combined total of 24,152 specimens were assessed, resulting in the evaluation of 5,046 *E. coli* and *Salmonella* spp. isolates. The overall pooled prevalence of *E. coli* and *Salmonella* spp. was 25.1% (95% CI: 18.9–32.5%) and 33.3% (95% CI: 29.3–37.5%), respectively. Lizards exhibited the highest pooled prevalence at 36.8% (95% CI: 21.5–55.4%), while snakes showed the lowest prevalence at 16.8% (95% CI: 8.7–29.9%). Regarding geographic distribution, the highest pooled prevalence occurred in Indonesia, reaching 98.7% (95% CI: 88.1–99.9%) for *Salmonella* spp. and an identical 98.7% (95% CI: 88.1–99.9%) for *E. coli*. Subgroup analysis by sample type revealed that the highest pooled prevalence of *Salmonella* spp. was documented in composite fecal samples, reaching 45.8% (95% CI: 32.5–59.8%). Conversely, for *E. coli*, the highest pooled estimate was derived from isolated fecal samples at 40.5% (95% CI: 7.6–84.9%). Methodological subgroup analysis revealed that the combination of culture and serotyping resulted in the highest pooled prevalence for *Salmonella* spp. at 48.1% (95% CI: 35.6–60.9%). On the contrary, the highest pooled prevalence of *E. coli* was 50.3% (95% CI: 32.2–68.3%) obtained from the studies using a combination of culture, biochemical testing and serotyping.

**Conclusion:**

A high global colonization burden of *Salmonella* spp. and *E. coli* exists in reptiles with significant variation by host taxonomy, geography and diagnostic method, and highlight the widespread occurrence of these bacteria in reptiles and their potential as zoonotic significance.

## 1 Introduction

Animals are colonized by microbiomes that play important roles in host metabolism and immune function (Colston and Jackson, 2016; Song et al., 2020). Reptiles are one of the most evolutionarily and ecologically diverse groups of vertebrates (Jacobson, 2007; Uetz et al., 2023) but were historically studied for their microbiota’s ability to transmit disease to humans (Lamm et al., 1972; Warwick et al., 2001). Although studies have begun to elucidate the diverse roles of reptilian gut microbiota (Siddiqui et al., 2022; Tremaroli and Bäckhed, 2012), it remains unclear if similar host gastrointestinal anatomy (cloaca) has led to conserved microbiome membership across species or if variation is driven by ecological differences at finer taxonomic resolutions.

Due to the increasing popularity of reptile ownership worldwide and subsequent enlargements of the human–animal interface, reptile-associated diseases now pose a greater threat to public health (Mendoza-Roldan et al., 2020; Pees et al., 2023). In fact, many reptiles associated bacteria that cause zoonotic disease are members of *Enterobacteriaceae* (*Salmonella*, *Escherichia*, *Klebsiella*, *Citrobacter*, *Proteus*, *Shigella*, Yersinia) (Lister and Barrow, 2009). These organisms have shown high prevalences on reptiles and reptile products throughout epidemiological investigations. Moreover, reptiles have been established as prominent reservoirs of *Salmonella* and are responsible for an estimated 6% of human salmonellosis cases of unknown origin (Mermin et al., 2004). Other reptile-associated zoonotic taxa that have been frequently detected include *Mycobacterium*, *Chlamydia*, *Leptospira*, and *Escherichia coli* sequence groups like B1 phylogroup (Ebani, 2017; Gordon and Cowling, 2003). However, the ecology and baseline prevalence of these enteric bacteria within the reptile gut microbiota is still heavily contested. *Salmonella* for example has been deemed an innocuous commensal of reptiles (Pasmans et al., 2021) despite prevalence ranging dramatically throughout host populations (Maciel et al., 2010) and contributing to acute disease syndromes (Clancy et al., 2016).

In fact, perhaps more worryingly comparisons between studies are difficult to even quantify, as culture independent molecular sampling techniques have shown *Salmonella* to be a less abundant or inconsistent member of reptile gut microbiota when compared to ISO standard culture techniques (Ahasan et al., 2018; Fagre et al., 2020; Zając et al., 2021). Other variables including captivity status, diet, temperature, and transmission routes also muddy our ability to understand *Salmonella*’ s persistence in gut communities when compared to other more stable reptilian commensals such as *E. coli* (Bestion et al., 2017; Holgersson et al., 2016).

While many Enterobacteriaceae are harmless commensal organisms of the gut microbiota, there are also many pathogens considered high consequence. For example, certain strains of *E. coli* can cause serious illness to both humans and animals. *Enterobacteriaceae* that have important ecological, veterinary, and public health significance include *Escherichia* (*E. coli*), *Salmonella*, and *Klebsiella* species, among others. To attempt to provide some context to these discrepancies we performed a systematic review and meta-analysis focused on reptiles to identify prevalence of *Salmonella* spp. and *E. coli* across studies. By better understanding the ecology of these bacteria within reptiles we can take a better informed and more holistic “One Health” approach to how they impact veterinary and human health.

## 2 Materials and methods

### 2.1 Systematic review and protocol registration

The objective of this review was to evaluate the global prevalence, serovar diversity, and distribution of *Salmonella* spp. and *Escherichia coli* across various reptile species from peer-reviewed journals published up to December 2025. This systematic review answers the question: What is the global prevalence of *Salmonella* spp. and *Escherichia coli* in reptiles, and how does prevalence vary according to reptile taxon, geographical region, sample type, and diagnostic method?

#### 2.1.1 Systematic review protocol

The study selection process was conducted in accordance with the Preferred Reporting Items for Systematic Reviews and Meta-Analyses (PRISMA) guidelines (Page et al., 2021; Ramatla et al., 2022), which have been confirmed on a check list (Supplementary Table S1). A comprehensive literature search was performed across relevant electronic databases to identify studies investigating gut microbiota in reptiles.

### 2.2 Database search strategy

A systematic literature search was performed across multiple electronic databases, including PubMed, Scopus, ScienceDirect, and Medline databases, for relevant articles published up to 30 December 2025, with the specific keywords: (Enterobacteriaceae [MeSH Terms] OR Salmonella OR “*E. coli*” OR “*Escherichia coli*”) AND (reptile OR reptilia OR snake OR lizard OR turtle OR tortoise OR testudines). Details regarding the search strings and keywords used are provided in Supplementary Table S2. A manual search of Google Scholar was performed to identify any further relevant studies. The last search took place on 03 March 2026. No attempt was made to obtain further information or retrieve unpublished studies from the authors of the original manuscripts.

### 2.3 Study selection

Selection for inclusion was conducted by three authors through a systematic review of titles and abstracts. After manual de-duplication, Microsoft Excel was used to screen titles and abstracts against the pre-specified inclusion criteria. Two reviewers (LM and TR) independently assessed all titles, abstracts, and selected full texts, with a third reviewer (JN) mediating any conflicts. Full-text articles were subsequently retrieved for all selected records and independently evaluated for eligibility. Studies were excluded if the full text could not be obtained or if missing data remained unavailable. In instances where multiple publications referred to the same study, the most comprehensive version was retained for analysis.

### 2.4 Inclusion and exclusion criteria

Records returned from searches were collated into bibliographic software and duplicates removed. Screening of retrieved literature was performed in two phases. Titles and abstracts were screened by two reviewers and assessed independently against eligibility criteria. Records that were unrelated to reptile hosts or did not assess target pathogens (*Salmonella* spp. and *E. coli*) were excluded. Full-text manuscripts were then retrieved for remaining articles and subjected to complete review. Articles had to be full-text, peer-reviewed, primary studies that reported on any order of reptile host. Studies had to explicitly detail methodology (microbiological, biochemical, molecular) by which detection of *Salmonella* spp. and/or *E. coli* from cloacal/fecal/intestinal samples was performed. Additionally, primary studies needed to report accessible, unabridged quantitative result data (sample sizes and total number of isolates) that could undergo formal meta-analysis. We excluded studies focused on non-reptilian hosts, evaluated non-target bacteria/viruses or reported invalid sample types. Reviews, commentaries and descriptive reports were also excluded if they did not report quantitative original frequencies. Individual datasets reporting prevalence from <10 individual reptiles were omitted to improve the robustness of our statistical estimates and account for bias of outlier sampling variability within small study populations.

### 2.5 Data extraction

A pre-piloted Microsoft Excel form was designed to standardize data collection, and data was extracted from each completed study by LM and verified by TR, ensuring uniformity, replicability, and accuracy of the collected data. Details that were subjected to interpretation during extraction were clarified by consensus. When necessary, the source publication was consulted. Information extracted included study details (first author, title, year of publication, country/region where the work was conducted, and length of sampling period), host details (general reptile group consisted of lizard, snake, turtle/tortoise, or crocodile, host common name, host family, and host species according to the Reptile Database to ensure consistent use of taxonomy), and isolate details [target organism *Salmonella* spp. or *E. coli*, matrix type, and detection method (culture, biochemical testing, serotyping, PCR, WGS or MALDI-TOF MS)]. If studies sampled more than one site such as multiple species, locations, or detection methods, those variables were split into separate rows to allow subgroup analysis. The specimen/sample was considered the appropriate denominator for this review because individual animal tracking was not typically reported for these retrospective datasets. Sample/specimen was chosen as the unit of analysis (analytical denominator) for this systematic review due to inconsistent reporting of individual animal-level data in the primary literature. Samples/specimens included cloacal/fecal, oral, and meat samples. As such, study-level prevalence estimates refer to the pooled proportion of positive samples per study population.

### 2.6 Quality assessment of included studies

The methodological quality of the included studies was assessed using the Joanna Briggs Institute (JBI) Critical Appraisal Checklist for Studies Reporting Prevalence Data (Khasapane et al., 2026), which was adapted for studies investigating microbiota in animal populations. The checklist includes nine parameters, of which eight were applicable to the studies included in this review. These criteria determined whether or not each study used an adequate sampling frame/method and sample size, as well as described the subjects/settings well (e.g., captive or wild-caught). Additional criteria assessed whether there was sufficient coverage of the identified sample for data analysis, whether valid methods were used for microbial identification, whether microbial composition was measured using standard and reliable methods, and whether appropriate statistical analyses were applied. Each criterion was scored as one point for a “yes” response and zero points for “no” or “unclear” responses, resulting in a maximum possible score of eight points. Based on the total score, studies were categorized as low quality (scores less than 4), moderate quality (scores between 4 and 6), or high quality (scores greater than 6). To reduce potential bias, two reviewers (LM and TR) independently conducted the quality assessment, and any discrepancies were resolved through discussion until consensus was reached.

### 2.7 Data synthesis and data analysis

Comprehensive Meta-Analysis (CMA), a meta-analysis of the prevalence of *Salmonella* spp. and *E. coli* in reptiles was performed using software v.4.0^1^ (Ramatla et al., 2026). Pooled prevalence of *E. coli* and *Salmonella* spp. isolates was conducted, and subgroup analysis was based on continent, country, year of publication, settings, and samples collected. Using the random effects model, forest plots with 95% confidence interval (CI) of study-specific effect sizes for the pooled prevalence were produced using random effects models. The heterogeneity among studies was assessed using *I^2^* statistic (Higgins et al., 2003). A value close to 0% indicates no heterogeneity, while a value close to 25%, 50 and 75% corresponds to low, moderate and high heterogeneity, respectively. To account for this statistical heterogeneity, tau-squared (*τ^2^*) was calculated to estimate the variance between the included studies within the random-effects model. The *p*-values correspond to the heterogeneities between studies from a Chi-squared test of the null hypothesis that there is no heterogeneity. Subgroups analysis was done based on sample/specimen type (cloacal/fecal samples, oral samples), diagnostic methods, and region. For each subgroup, pooled prevalence was estimated using random-effects model. Statistical significance of difference between subgroups was determined by Q-test of heterogeneity among groups. *p* < 0.05 was considered statistically significant.

### 2.8 Publication bias

Publication bias was measured using funnel plots to test for symmetry, and this was further complemented using the Beg and Mazumdar rank correlation test and Egger’s regression test. For all the tests, a *p*-value of < 0.05 was considered statistically significant (Begg and Mazumdar, 1994; Ramatla et al., 2023).

## 3 Results

### 3.1 Literature search and eligible studies

The first systematic database search revealed a total of 2,124 records from five electronic databases, including PubMed, Google Scholar, ScienceDirect, MEDLINE, and Scopus. We first screened 1,577 unique citations after excluding 547 duplicate records based on titles and abstracts. The outcome of this screening stage was the exclusion of 1,249 papers that were not suitable. This left 328 full-text articles that were formally assessed for eligibility. Of these, 139 full-text articles were excluded based on strict inclusion and exclusion criteria, leading to a final pool of 189 eligible studies included in the meta-analysis (Figure 1; Supplementary Table S3). Methodological quality was assessed using the JBI Critical Appraisal Checklist with a scoring range of 1–8. The minimum quality score for inclusion of studies was set at 50%. However, all 189 papers evaluated met this benchmark, with a final score of greater than 5, fulfilling the eligibility criteria established by Moola et al. (2015) (Supplementary Table S4).

**Figure 1:**
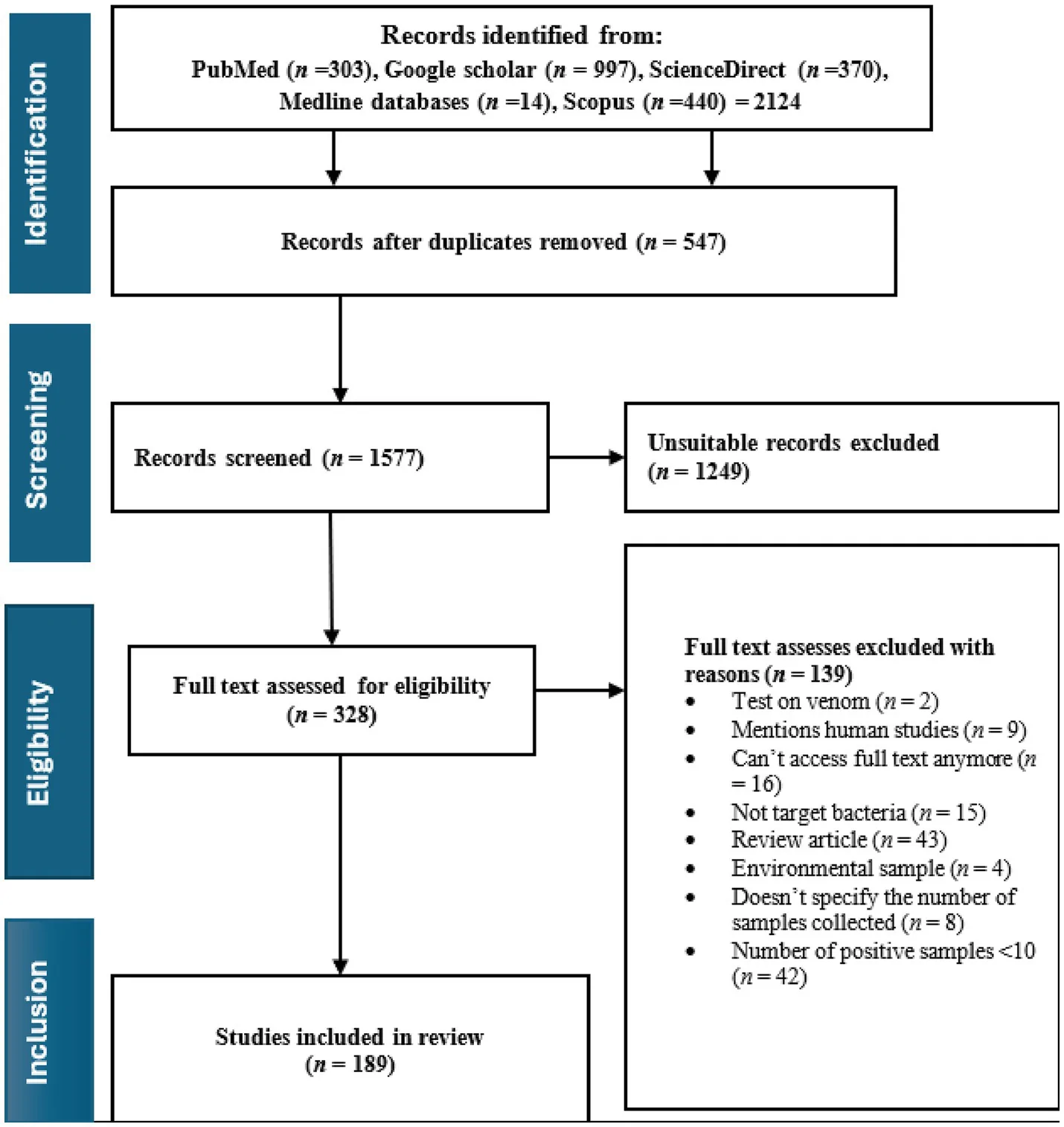
The PRISMA diagram shows the study selection process.

### 3.2 Distribution of the articles according to continents

The 189 studies included in this systematic review were published from 1968 up to 2025, with most publications appearing after 2000. Studies were conducted across seven geographic categories, comprising six continents and an additional “other” (with more than one location) category for studies that did not fit within a single continental classification. Europe contributed the largest number of studies (*n* = 59), followed by Asia (*n* = 38), North America (*n* = 38), South America (*n* = 23), Africa (*n* = 15), Oceania (*n* = 14), and the “other” category (*n* = 2) (Figure 2). Collectively, the included studies examined 24,152 individual reptiles. Europe also accounted for the highest number of reptiles sampled (*n* = 7,091), followed by Asia (*n* = 6,091), North America (*n* = 3,906), Oceania (*n* = 3.667), South America (*n* = 1,765), Africa (*n* = 1,367), and the “other” category (*n* = 265).

**Figure 2:**
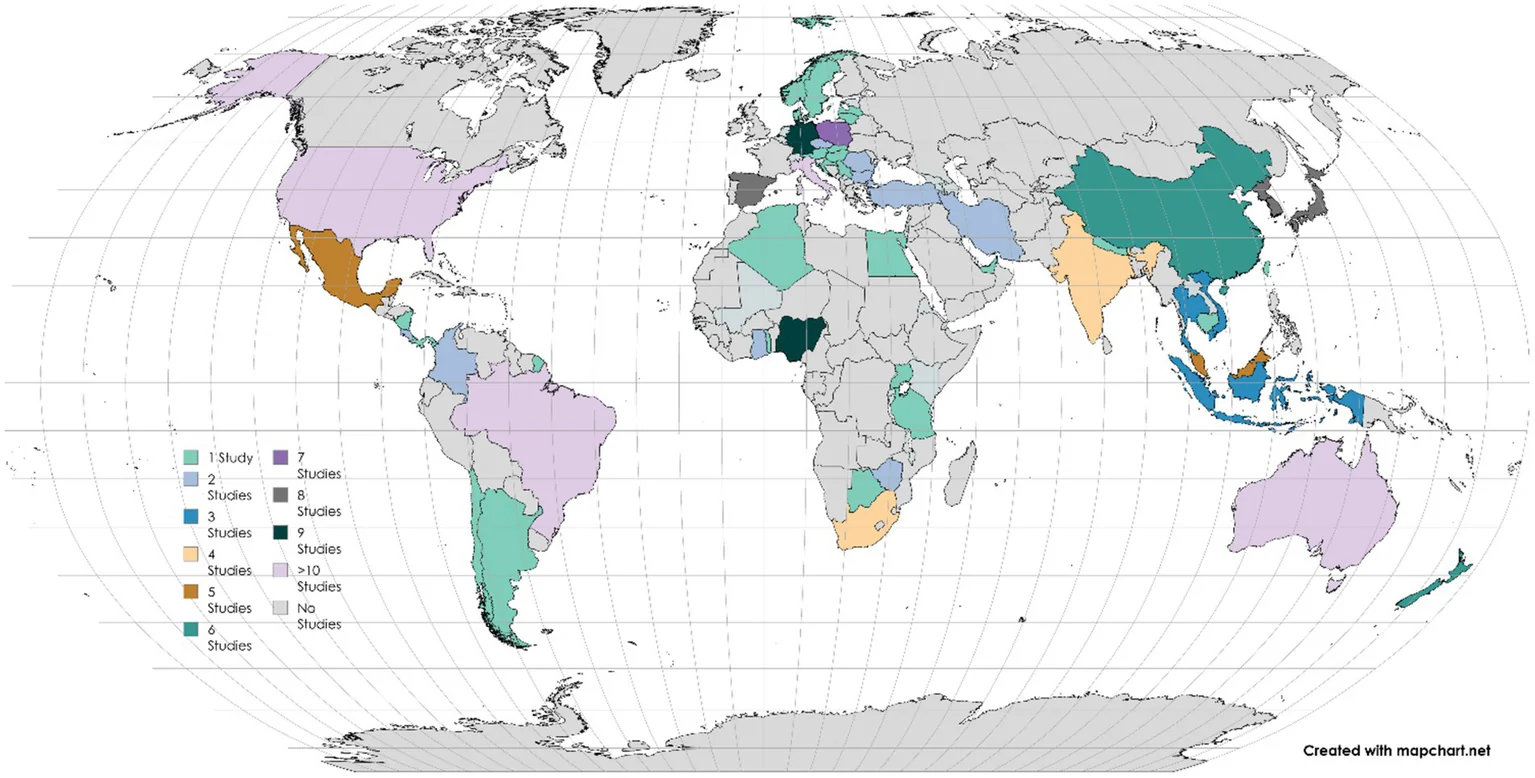
World map showing the number of articles from different countries that reported the global diversity of *Salmonella* spp. and *Escherichia coli* in reptiles (https://www.mapchart.net/world.html, accessed on May 6, 2026).

### 3.3 Distribution of *Salmonella* and *E. coli* across reptile taxa

Examining positivity across reptile groups, *Salmonella* spp. was identified in 50.9% (1,571/3086) of snakes tested, whereas *E. coli* was present in 17.7% (182/1026) of snakes. *Salmonella* spp. was identified in 28.0% (3,055/10926) of lizards, whereas *E. coli* was identified most frequently in this group at 38.1% (821/2154). In crocodilians, 29.6% (271/916) of samples were positive for *Salmonella* and 24.7% (132/534) of samples harbored *E. coli*. Lastly, chelonians had a *Salmonella* spp. prevalence of 23.4% (1,343/5744) and 27.5% (315/1145) positivity for *E. coli*.

### 3.4 Distribution of articles according to reptile species

The reptile taxa included in the reviewed studies represented a broad range of species, with chelonians being the most frequently investigated group, comprising 35% of all sampled reptiles (*n* = 86). Lizards were the second most commonly studied group, accounting for 32% of the total sample (*n* = 80), followed by snakes, which represented 28% (*n* = 69). Crocodilians were the least represented group, constituting only 5% of the sampled reptiles (*n* = 13).

Chelonians were further categorized into several subgroups, including studies that referred to specimens generally as chelonians (*n* = 7), as well as more specific classifications such as turtles (*n* = 50), tortoises (*n* = 23), and terrapins (*n* = 12). Similarly, lizards were subdivided into studies that identified specimens broadly as lizards (*n* = 46) and those focusing on specific taxa, including iguanas (*n* = 14) and geckos (*n* = 16). The majority of included studies originated from captive (*n* = 87, 46%) and wild-caught (*n* = 70, 37%) animal populations. A smaller proportion used mixed sources (*n* = 19, 10%), temporary captive/rehab/rescue individuals (*n* = 9, 4%), while the source population was unclear in only 4 studies (2%) (Figure 3).

**Figure 3:**
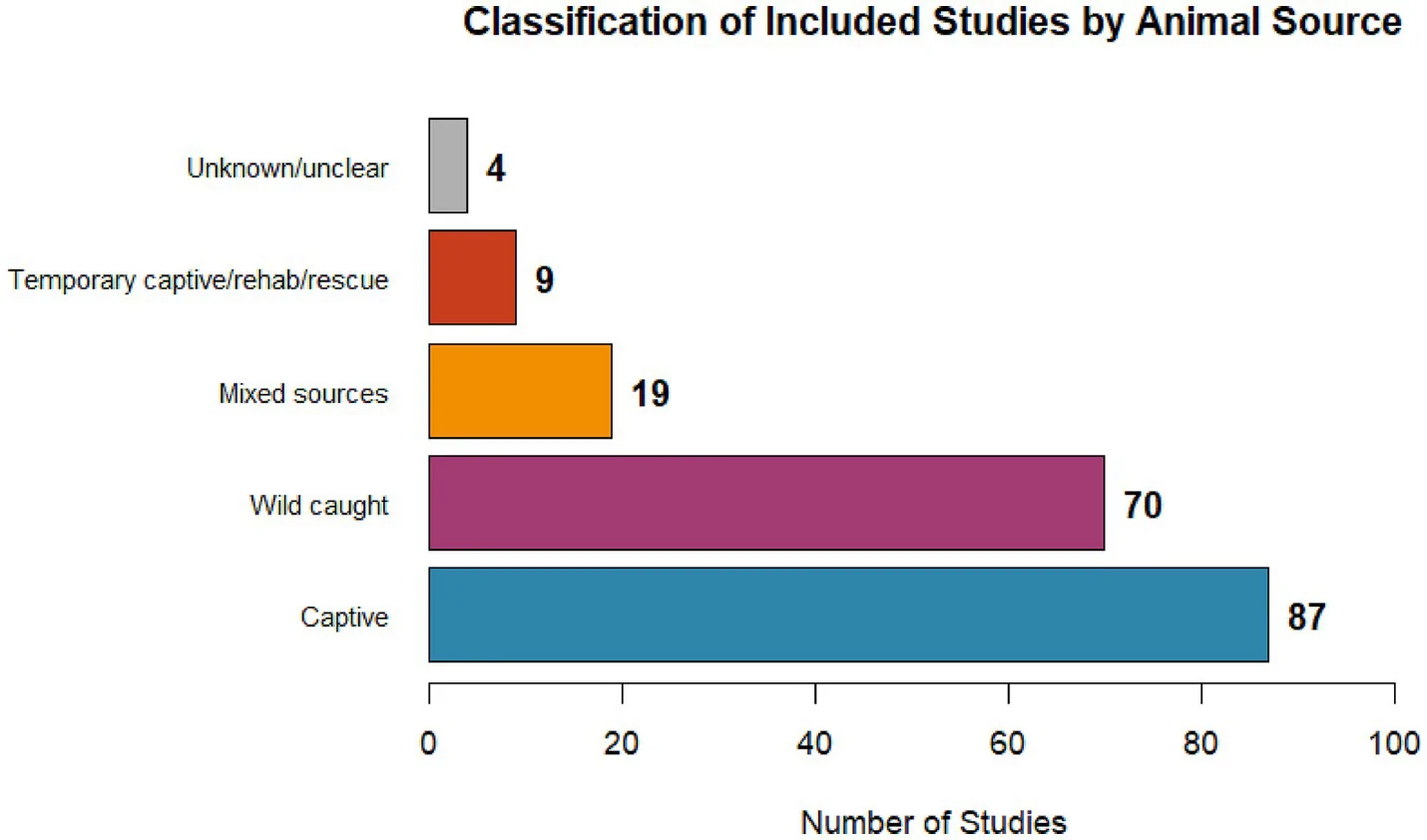
Distribution of included studies (*n* = 189) by source population of the animals. Categories were assigned based on the descriptions provided in the original studies. Captive refers to animals maintained under long-term human care. Temporary captive/rehab/rescue includes animals in short-term holding for rehabilitation, rescue, or temporary research procedures prior to release or transfer. Wild caught denotes animals sampled directly from free-ranging populations. Mixed sources indicate studies that included animals from multiple origin categories that could not be reliably attributed to a single group. Studies were classified as Unknown/unclear if the origin was not explicitly stated or was ambiguous.

Overall, the reviewed studies encompassed a taxonomically diverse set of reptile hosts, although the distribution was skewed toward chelonians and lizards, which together accounted for more than two-thirds of all sampled animals. Cloacal/fecal samples were the most frequently collected specimen type, accounting for 126 studies. This was followed by cloacal swabs (*n* = 18) and another category (*n* = 18), which comprised a variety of less commonly used sample types, including tissue, and blood samples. Fecal samples alone were used in 13 studies, while both oral and cloacal swabs were collected in 8 studies and the oral swabs were used in 6 studies.

A variety of bacterial detection and identification methods were employed across the reviewed studies. The most used approach combined culture, biochemical characterization, and serotyping, which was reported in 96 (49%) studies. Culture followed by biochemical identification alone was used in 47 (24%) studies. Culture in combination with polymerase chain reaction (PCR) was employed in 19 (10%) studies, while 15 (8%) studies utilized other methodologies, including molecular and immunological techniques not captured within the major categories. Less frequently used approaches included culture with serotyping (*n* = 9; 5%) and culture, serotyping, and PCR (*n* = 7; 4%). Advanced analytical methods were rarely applied, with culture coupled to matrix-assisted laser desorption/ionization time-of-flight mass spectrometry (MALDI-TOF MS) reported in 2 studies (1%), and culture followed by whole-genome sequencing (WGS) and genomic analysis also reported in 2 (1%) studies.

### 3.5 Meta-analysis results on overall prevalence: *E. coli* and *Salmonella* spp. in reptiles

The pooled prevalence estimates for colonization by *E. coli* and *Salmonella* spp. in reptiles and their respective taxonomic subgroups are summarized in Table 1. The Meta-Analysis estimated the overall pooled prevalence of *E. coli* colonization in reptiles to range between 25.1% (95% CI: 18.9–32.5%, *τ^2^* = 1.578, *I^2^* = 94.9%), indicating considerable variability among the included studies. While *Salmonella* spp. colonization in reptiles was estimated at 33.3% (95% CI: 29.3–37.5%, *τ^2^* = 1.110, *I^2^* = 96.1%). *Salmonella* spp. is about 8.2% more common in reptiles overall than *E. coli*. The confidence interval for *Salmonella* spp. is narrower (8.2%) indicating a more precise estimate than that for *E. coli* (13.6%). Both estimates have high (*I^2^*) percentages, indicating that almost all of the observed variance is due to real differences between studies and not sampling error.

**Table 1:** Pooled prevalence estimates and 95% CI of *Salmonella* spp. and *Escherichia coli* in reptiles.

Risk factors	Number of studies	Pooled estimates			Measure of heterogeneity			
		Sample size	Positive isolates	I^2^ (95%CI)	Q-Value	I^2^	*df* [Q]	*p*-value
*E. coli*	53	5,046	1919	25.1 (18.9–32.5)	1019.531	94.9	52	0.326
*Salmonella* spp.	160	22,842	7,230	33.3 (29.3–37.5)	4125.237	96.1	159	0.403
Snakes								
*E. coli*	16	1,026	182	16.8 (08.7–29.9)	183.990	91.8	15	0.752
*Salmonella* spp.	53	3,086	1,571	49.4 (40.1–58.9)	686.275	92.4	52	0.314
All lizards								
*E. coli*	16	2,154	821	36.8 (21.5–55.4)	455.744	96.7	15	0.752
*Salmonella* spp.	64	10,926	3,055	34.5 (28.8–40.7)	1527.526	95.8	63	0.256
Crocodile								
*E. coli*	4	534	132	25.3 (10–50.8)	43.090	93.03	3	0.496
*Salmonella* spp.	9	916	271	29.9 (17.9–45.5)	112.738	92.9	8	0.531
Chelonians								
*Salmonella* spp.	7	613	43	06.6 (03.0–14.0)	34.170	82.4	6	0.452
All chelonians								
*E. coli*	20	1,145	315	21.9 (14.1–32.3)	151.904	87.4	19	0.745
*Salmonella* spp.	66	5,744	1,343	23.2 (17.9–29.4)	1114.514	94.168	65	0.794
Lizards								
*E. coli*	5	673	199	48.3 (18.5–79.4)	135.963	97.058	4	0.141
*Salmonella* spp.	41	6,408	1850	35.5 (28.3–43.4)	950.740	95.793	40	0.196
Iguanas								
*E. coli*	4	400	297	56.5 (31.7–78.5)	25.562	88.264	3	0.496
*Salmonella* spp.	10	426	248	50.0 (36.4–63.6)	35.953	74.968	9	0.654
Gecko								
*E. coli*	4	774	172	15.1 (03.5–46.4)	133.815	97.758	3	1.000
*Salmonella* spp.	12	2,802	770	29.7 (19.6–42.4)	304.450	96.387	11	0.583
Turtles								
*E. coli*	12	855	94	14.2 (08.6–22.5)	65.304	83.156	11	0.583
*Salmonella* spp.	38	3,640	831	24.7 (18.2–32.7)	618.150	94.014	37	0.641
Tortoise								
*E. coli*	6	252	53	24.9 (07.1–58.8)	57.405	91.290	5	0.850
*Salmonella* spp.	17	1,135	281	23.4 (15.1–34.5)	135.301	88.174	16	0.564
Terrapin								
*E. coli*	5	230	199	41.1 (18.1–68.9)	39.114	89.774	4	0.624
*Salmonella* spp.	7	495	232	26.7 (06.3–66.3)	173.217	96.536	6	0.652

### 3.6 Prevalence by reptile taxon

The prevalence of *E. coli* varied across the major reptile taxa (Table 1). Lizards exhibited the highest pooled prevalence at 36.8% (95% CI: 21.5–55.4%, *τ^2^* = 2.028, *I^2^* = 96.7%), followed by crocodilians at 25.3% (95% CI: 10.0–50.8%; *τ^2^* = 1.174, *I^2^* = 93.0%). Chelonians had a pooled prevalence of 21.9% (95% CI: 14.1–32.3%; *τ^2^* = 1.193, *I^2^* = 87.4%) (Figure 4), while snakes showed the lowest prevalence at 16.8% (95% CI: 8.7–29.9%; *τ^2^* = 1.951, *I^2^* = 91.8%). Within the lizard subgroup, iguanas had the highest pooled prevalence of *E. coli* colonization at 56.5% (95% CI: 31.7–78.5%; *τ^2^* = 0.712, *I^2^* = 88.2%), followed by studies reporting unspecified lizard species at 48.3% (95% CI: 18.5–79.4%; *τ^2^* = 2.486, *I^2^* = 97.0%). Geckos exhibited a lower prevalence of 15.1% (95% CI: 3.5–46.4%; *τ^2^* = 2.331, *I^2^* = 97.7%). Among chelonians, terrapins showed the highest prevalence at 41.1% (95% CI: 18.1–68.9%; *τ^2^* = 1.401, *I^2^* = 89.7%), followed by tortoises at 24.9% (95% CI: 7.1–58.8%; *τ^2^* = 2.796, *I^2^* = 91.2%). Turtles had the lowest prevalence within this group at 14.2% (95% CI: 8.6–22.5%; *τ^2^* = 0.800, *I^2^* = 83.1%). Tortoises had the least precise estimate with a large 95% CI of 51.7% (7.1 to 58.8%). Within the specific subgroups, turtles have the lowest overall variance (*τ^2^* = 0.800) and the smallest 95% CI range (13.9%). Very high *I^2^* values for all classifications indicate that the large variation among studies is not fully explained by stratification of subgroups. Among the major reptile taxa, snakes had the highest pooled prevalence of *Salmonella* spp. at 49.4% (95% CI: 40.1–58.9%; *τ^2^* = 1.622, *I^2^* = 92.4%). Lizards followed with a prevalence of 34.5% (95% CI: 28.8–40.7%; *τ^2^* = 0.990, *I^2^* = 95.8%), while chelonians exhibited lower a prevalence of 23.2% (95% CI: 17.9–29.4%; *τ^2^* = 1.528, *I^2^* = 94.1%).

**Figure 4:**
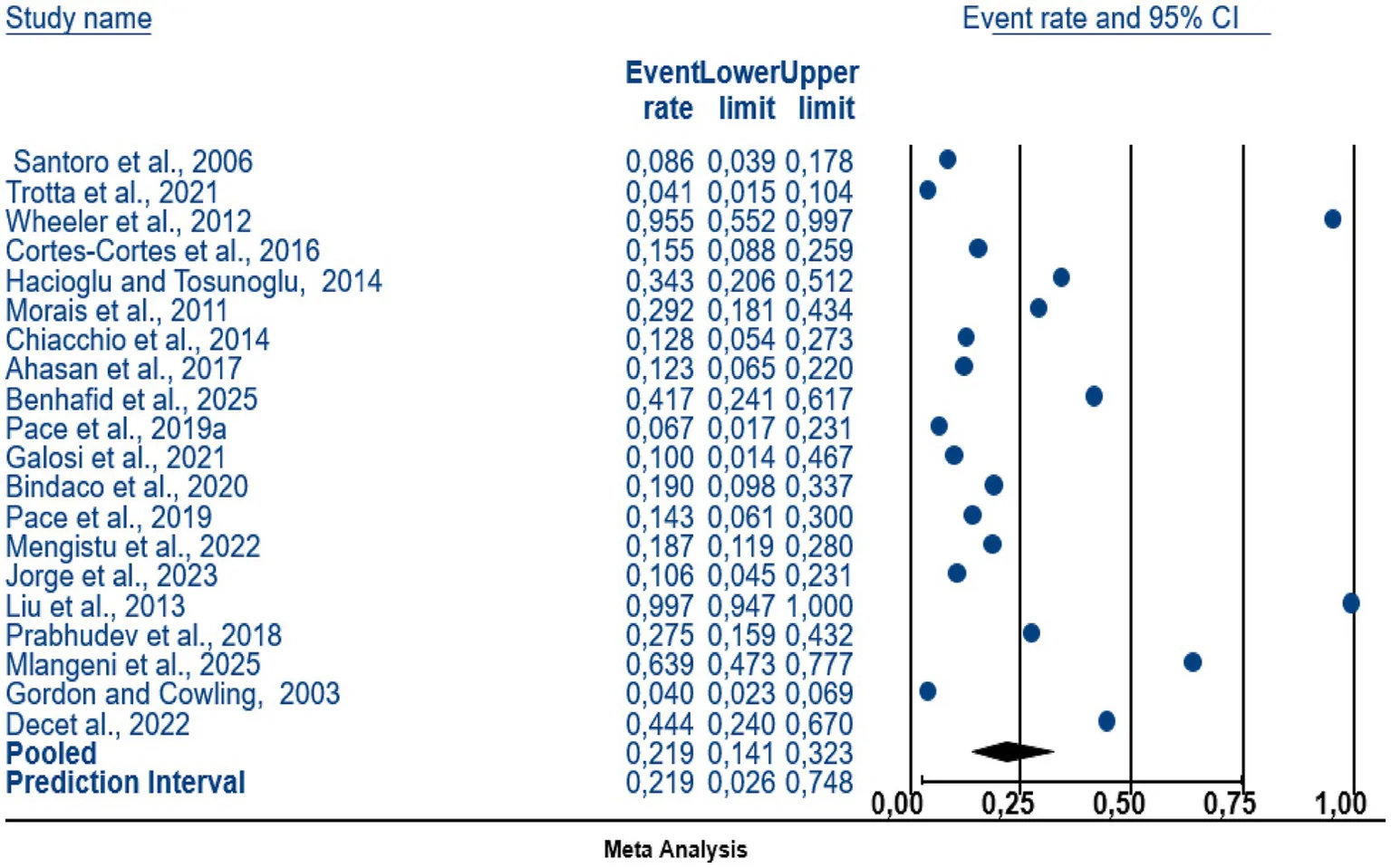
Forest plot showing the pooled prevalence of chelonians. The squares show the individual point estimate. The diamond at the base indicates the pooled estimates from the total studies.

Within the lizard subgroup, iguanas demonstrated the highest pooled prevalence of *Salmonella* spp. at 50.0% (95% CI: 36.4–63.6%; *τ^2^* = 0.489, *I^2^* = 74.96%), followed by unspecified lizard species at 35.5% (95% CI: 28.3–43.4%; *τ^2^* = 1.018, *I^2^* = 95.79%). Geckos had a pooled prevalence of 29.7% (95% CI: 19.6–42.4%; *τ^2^* = 0.868, *I^2^* = 96.38%). Among chelonians, terrapins exhibited the highest prevalence at 26.7% (95% CI: 18.1–68.9%; *τ^2^* = 4.823, *I^2^* = 96.53%), followed by turtles at 24.7% (95% CI: 18.2–32.7%; *τ^2^* = 1.300, *I^2^* = 94.01%) and tortoises at 23.4% (95% CI: 15.1–34.5%; *τ^2^* = 0.897, *I^2^* = 88.17%). Studies classified broadly as chelonians reported the lowest prevalence at 6.6% (95% CI: 3.0–14.0%; *τ^2^* = 0.979, *I^2^* = 82.4%) (Table 1). For the *E. coli* and *Salmonella* spp. data, the lowest *I^2^* value (74.9%) is for the iguanas, so the studies on them are the most consistent. Between-study variance was very high for terrapins (*τ^2^* = 4.823). The 95% CI is highly asymmetric and wide (50.8%). *Salmonella* spp. is much more likely to be found in geckos (29.7%) than *E. coli* (15.1%).

### 3.7 *Salmonella enterica* subspecies and serovars and zoonotic strains

*Salmonella enterica* comprised 99.96% of *Salmonella* infections; *S. enterica* subsp. *enterica* (I) was the most common infective subspecies (68.5%), followed by *S*. diarizonae subsp. diarizonae (IIIb), *S*. houtenae subsp. houtenae (IV), *S*. salamae subsp. salamae (II) and *S. arizonae* subsp. arizonae (IIIa). *Salmonella enterica* subsp. *enterica* (I) was the most common subspecies isolated from turtles and lizards while *S*. diarizonae subsp. diarizonae was the predominant subspecies infecting snakes. Over 500 serovars were identified throughout studies, and serovar diversity was higher among captive animals. Captive animals had a greater diversity of serovars than their wild counterparts. The global pooled prevalence of *S. enterica* subsp. *enterica* (I) among reptiles was estimated to be 35.1% (95% CI: 31.4–39.1%). Overall, prevalence was highest among snakes (45.3%) and lizards (38.5%). Although reptiles from captive facilities were more likely to test positive for *Salmonella* spp. in general, no significant difference was seen between wild and captive groups for overall prevalence of subsp. *enterica*.

The analysis established that host-generalist zoonotic strains had the greatest weighting among pooled reptile groups, with an overall pooled prevalence of 18.4% (95% CI: 14.1 - 23.6%). *S. enterica* subspecies *enterica* (I) serovars made up the greatest portion of zoonotic strains, and unsurprisingly, consistently resulted in the highest pooled prevalence estimates such as *S. typhimurium*, *S. enteritidis*, *S*. Newport, *S*. Saintpaul, *S*. Agona, *S. infantis*, and *S*. Javiana. Non-zoonotic or cold-blooded strains; *Salmonella enterica* subspecies *salamae* (II), *arizonae* (IIIa), *diarizonae* (IIIb), and *houtenae* (IV) had a lower pooled prevalence of 5.9% [95% CI (3.8%, 9.1%)]. While rare in human clinical settings, underlying data branches on the CMA forest plot for these cold-blooded lineages such as *S*. Hermannswerder, *S*. Langford, and specialized *S. diarizonae* formulas, demonstrated notable host-specific clustering within snake and turtle subgroups. Although subspecies *salamae* (II), *arizonae* (IIIa), *diarizonae* (IIIb), and *houtenae* (IV) are traditionally less common in human clinical cases, their pronounced host-specific clustering within snake and turtle subgroups represents an under-reported environmental burden.

### 3.8 Subgroup analysis by continents

Studies investigating the prevalence of *Salmonella* spp. in reptiles were conducted across six continents, and the pooled prevalence estimates by geographic region are presented in Table 2. Among the included regions, Asia reported the highest pooled prevalence of *Salmonella* spp. colonization at 40.6% (95% CI: 31.3–50.6%; *τ^2^* = 1.166, *I^2^* = 96.38%), closely followed by Africa at 38.4% (95% CI: 25.7–52.9%; *τ^2^* = 0.824, *I^2^* = 91.73%). South America exhibited a pooled prevalence of 37.5% (95% CI: 24.4–52.6%; *τ^2^* = 1.511, *I^2^* = 94.19%), while North America and Europe showed prevalence estimates of 32.5% (95% CI: 24.0–42.4%; *τ^2^* = 1.310, *I^2^* = 94.66%) and 31.0% (95% CI: 25.1–37.7%; *τ^2^* = 1.038, *I^2^* = 95.5%), respectively. The lowest pooled prevalence was reported in Oceania, with an estimate of 19.9% (95% CI: 8.6–39.6%; *τ^2^* = 2.758, *I^2^* = 98.7%). The prevalence of *E. coli* colonization in reptiles was similarly evaluated across six continents, with pooled prevalence estimates summarized in Table 2. Oceania recorded the highest pooled prevalence of *E. coli* at 57.4% (95% CI: 15.4–90.9%; *τ^2^* = 3.008, *I^2^* = 96.9%), followed by North America at 44.9% (95% CI: 19.7–73.0%; *τ^2^* = 2.171, *I^2^* = 94.7%). Europe exhibited the lowest pooled prevalence of *E. coli* colonization, estimated at 13.2% (95% CI: 6.4–25.5%; *τ^2^* = 2.084, *I^2^* = 95.4%).

**Table 2:** Subgroup analysis of *Salmonella* spp. and *E. coli* in reptiles reported worldwide stratified by continents.

Continents	Number of studies	Sample size	Positive isolates	I^2^ (95%CI)	Q-value	I^2^	*df* [Q]	*p*-value
Africa								
*Salmonella* spp.	12	1,289	371	38.4 (25.7–52.9)	133.098	91.735	11	0.039
*E. coli*	8	832	175	31.5 (16.2–52.3)	102.941	93.200	7	1.000
Asia								
*Salmonella* spp.	32	5,575	1856	40.6 (31.3–50.6)	858.088	96.387	31	0.445
*E. coli*	14	967	428	34.4 (16.3–58.4)	204.990	93.658	13	0.584
Europe								
*Salmonella* spp.	52	6,676	2,321	31.0 (25.1–37.7)	1145.898	95.549	51	0.564
*E. coli*	14	1,383	277	13.2 (06.4–25.5)	286.343	95.460	13	0.207
North America								
*Salmonella* spp.	34	3,478	1,062	32.5 (24.0–42.4)	618.474	94.664	33	0.863
*E. coli*	7	555	429	44.9 (19.7–73.0)	113.945	94.734	6	0.880
Oceania								
*Salmonella* spp.	12	3,915	877	19.9 (08.6–39.6)	858.333	98.718	11	0.890
*E. coli*	3	459	238	57.4 (15.4–90.9)	64.987	96.922	2	0.601
South America								
*Salmonella* spp.	18	1,397	532	37.5 (24.4–52.6)	292.883	94.196	17	0.569
*E. coli*	11	787	355	33.4 (18.4–52.8)	170.647	94.140	10	0.311

An important geographical pattern observed in this meta-analysis was the contrasting prevalence estimates for *Salmonella* spp. and *Escherichia coli* in Oceania. It has the lowest rate of *Salmonella* spp. colonization in the world (19.9%) but the highest rate of *E. coli* (57.4%). The estimate for *E. coli* in Oceania is highly unstable, with a huge 95% CI spread (75.5%, from 15.4 to 90.9%) and the largest overall variance (*τ^2^* = 3.008). Across all six continents and both pathogens, (*I^2^*) remains high (>91%) confirming that geographic region in and of itself does not resolve the statistical variance between study methodologies.

### 3.9 Subgroup analysis by country

Country-level subgroup analyses revealed substantial variation in the pooled prevalence estimates of both *Salmonella* spp. and *Escherichia coli* among reptiles as shown in Supplementary Table S5. Countries represented by a single study were excluded from meta-analysis. For *Salmonella* spp., the highest pooled prevalence was reported in Indonesia at 98.7% (95% CI: 88.1–99.9; *τ^2^* = 1.942, *I^2^* = 63.18%), followed by Thailand at 91.1% (95% CI: 2.5–100.0; *τ^2^* = 17.693, *I^2^* = 94.58%). The lowest prevalence estimates were observed in Trinidad (14.4%; 95% CI: 10.1–20.2; *τ^2^* = 0.0, *I^2^* = 0.0%) and New Zealand (4.4%; 95% CI: 2.2–8.6; *τ^2^* = 10.581, *I^2^* = 91.5%). For *E. coli*, Indonesia again exhibited the highest pooled prevalence estimate at (98.7%; 95% CI: 88.1–99.9; *τ^2^* = 1.942, *I^2^* = 63.1%), followed by Ecuador (86.8%; 95% CI: 33.4–98.8; *τ^2^* = 2.657, *I^2^* = 71.6%) and South Africa (79.2%; 95% CI: 29.5–97.2, *τ^2^* = 1751, *I^2^* = 60.8%). The lowest pooled prevalence estimates were reported in Malaysia (10.5%; 95% CI: 5.1–20.4; *τ^2^* = 0.0, *I^2^* = 0.0%), Italy (4.8%; 95% CI: 2.2–10.3; *τ^2^* = 0.0, *I^2^* = 0.0%), and Bulgaria (3.6%; 95% CI: 1.5–8.4; *τ^2^* = 0.0, *I^2^* = 0.0%).

Heterogeneity analyses revealed perfect statistical consistency across specific national cohorts. In the pools for Trinidad (*Salmonella* spp.), Malaysia (*E. coli*), Italy (*E. coli*), and Bulgaria (*E. coli*), both the *I^2^* and *τ^2^* statistics reached 0.0%. This shows a perfect statistical consistency across studies pooled in these specific countries. The estimate for *Salmonella* spp. in Thailand is very unstable with an extreme variance (*τ^2^* = 17.693) and a confidence interval stretching from almost the very lowest (2.5%) to the very highest (100%) percentages. Data are the same for both pathogens for Indonesia. This indicates either a very high true baseline rate for the enteric bacteria in the sampled reptile populations, or a common data structure between the two analyses.

### 3.10 Subgroup analysis by sample type

The pooled prevalence estimates for *Salmonella* spp. colonization in reptiles, stratified by the type of biological sample collected, are detailed in Supplementary Table S6. A marked heterogeneity in prevalence was evident across the different sample modalities. The highest pooled prevalence was documented in composite fecal samples alone, which demonstrated a prevalence of 45.8% (95% CI: 32.5–59.8%; *τ^2^* = 0.493, *I^2^* = 92.1%). Lower colonization frequencies were observed in combined oral and cloacal swabs (13.4%; 95% CI: 7.2–23.6%; *τ^2^* = 0.456, *I^2^* = 76.4%). All modalities have high (*I^2^*) values (76.4 to 95.7%), reflecting severe statistical heterogeneity among pooled studies. When using the combination of direct animal shedding (cloacal/fecal) and the immediate environment, the pooled prevalence estimate is drastically increased compared to testing individual animal sites only.

The pooled prevalence estimates for *E. coli* colonization in reptiles, disaggregated by sample type, are also presented in Supplementary Table S6. Considerable variation in prevalence was observed depending on the nature of the specimen collected. The highest estimate was derived from fecal samples at 40.5% (95% CI: 7.6–84.9%; *τ^2^* = 6.606, *I^2^* = 97.3%). Cloacal/fecal samples followed, with a pooled prevalence of 31.8% (95% CI: 22.3–43.2%; *τ^2^* = 1.402, *I^2^* = 94.7%). The oral swab sample types yielded lower prevalence of 18.0% (95% CI: 10.9–28.3%; *τ^2^* = 0.081, *I^2^* = 17.3%).

### 3.11 Subgroup analysis by detection method

Overall, prevalence estimates varied considerably depending on the laboratory methods utilized. The highest pooled prevalence of *Salmonella* spp. was observed when culture was combined with serotyping at 48.1% (95% CI: 35.6–60.9%; *τ^2^* = 0.483, *I^2^* = 88.3%) (Supplementary Table S6). This was followed by the combined culture, biochemical, and serotyping approach, which yielded a prevalence of 35.5% (95% CI: 29.9–41.4%; *τ^2^* = 1.313, *I^2^* = 96.9%). Lower pooled prevalence estimates were obtained using culture with MALDI-TOF MS (8.9%; 95% CI: 2.1–30.1%, *τ^2^* = 1.00, *I^2^* = 85.94%). Marked variation was similarly observed across diagnostic approaches. The highest pooled prevalence of *E. coli* was reported for studies which used a combination of culture, biochemical testing, and serotyping at 50.3% (95% CI: 32.2–68.3%; *τ^2^* = 0.713, *I^2^* = 91.56%). This was followed by culture with biochemical identification, which yielded a prevalence of 23.1% (95% CI: 16.6–31.2%; *τ^2^* = 1.042, *I^2^* = 90.46%). Lower prevalence estimates were observed for culture combined with PCR (14.9%; 95% CI: 6.0–32.5%; *τ^2^* = 2.405, *I^2^* = 95.37%), while the lowest prevalence was reported for culture combined with MALDI-TOF MS at 3.9% (95% CI: 1.9–7.5%, *τ^2^* = 0.0, *I^2^* = 0.0%).

The *E. coli* culture + MALDI-TOF MS subgroup showed zero statistical heterogeneity (*I^2^* = 0.0%, *τ^2^* = 0.0), implying little noise between studies due to limited cross-reactivity of high-specificity mass spectrometry metabolomic profiles. By comparison, multi-step diagnostic tests had significantly higher pooled prevalences for both organisms likely because they detect a wider range of phenotypes but add more noise between studies. Additionally, the reported between-study variance was extreme for PCR-based detection of *E. coli* (*τ^2^* = 2.405), implying noisy data. This is most likely due to lab-specific practices such as target gene variation such as *uidA* vs. 16S rRNA, differences in DNA extraction rates, and ability of the assay to amplify DNA from dead target cells.

### 3.12 Meta-regression analysis over time

Meta-regression of *Salmonella* spp. prevalence data revealed a highly significant, strong positive linear trend between year of publication and reported prevalence (*F* (1, 161) = 103.40, *p* < 0.001, *R^2^* = 0.391). The slope was positive (*β* = 1.64 40, SE = 0.162, *t* = 10.17, *p* < 0.001, 95% CI: 1.32 48 to 1.96 33), reflecting an estimated average annual increase in *Salmonella* spp. colonization of (1.64%) throughout the examined timeframe. Meta-regression of *E. coli* prevalence data demonstrated a highly significant, moderate positive relationship between year of publication and reported prevalence (*F* (1, 55) = 11.31, *p* = 0.00 14, *R^2^* = 0.171). Year had a positive effect on prevalence in this regression as indicated by the positive slope *β* = 0.88 45, SE = 0.263, t = 3.36, *p* = 0.00 14, 95% CI: 0.35 73 to 1.4118), with estimates suggesting a significant annual increase of (0.88%). A formal Z-test was used to compare the regression coefficients of these two regressions. The predicted prevalence of *Salmonella* spp. increased significantly faster than predicted prevalence of *E. coli* (Δβ = 0.7595, *SE_diff_* = 0.30 89, Z = 2.459, *p* = 0.0139, nearly twice as fast on an annual basis.

### 3.13 Risk of publication bias of included studies

Publication bias was assessed using funnel plot analysis to evaluate the symmetry of the included studies, and this was further complemented by the Begg and Mazumdar rank correlation test as well as Egger’s regression test. For all analyses, *p* < 0.05 was considered statistically significant. However, statistically significant asymmetry was detected for studies investigating *E. coli* in cloacal samples (*p* = 0.010) (Supplementary Figure S1a) and for *Salmonella* spp. studies conducted in Africa (*p* = 0.039) (Supplementary Figure S1b), indicating the possible presence of publication bias within these subgroups.

## 4 Discussion

To our knowledge, this study represents the first global systematic review and meta-analysis to jointly estimate the prevalence of both *Salmonella* spp. and *Escherichia coli* in reptiles. Using a systematic approach, we identified and synthesized evidence from 189 peer-reviewed studies conducted on six continents, covering a total of 24,152 individual reptiles. The pooled prevalence data from diverse geographic regions, reptile taxa, and study settings, we generated pooled global prevalence estimates and evaluated the distribution of these bacterial pathogens among reptilian hosts around the world.

*Escherichia coli* is a common inhabitant of the gastrointestinal tract of warm-blooded vertebrates but has also been reported in ectothermic animals, including reptiles (Gordon and Cowling, 2003; Mlangeni et al., 2025). The occurrence of *E. coli* in reptiles can be influenced by factors such as diet, environmental exposure, husbandry conditions, and interactions with other animals (Pantuzza Ramos et al., 2019). In the present study, the pooled prevalence of *E. coli* was estimated at 25.1% (95% CI: 18.9–32.5%), indicating that approximately one-quarter of sampled reptiles harbored this bacterium. To our knowledge, this is the first meta-analysis to provide a global estimate of *E. coli* prevalence in reptiles, limiting direct comparisons with previous reptile-focused syntheses. However, Assefa and Bihon (2018) reported a pooled prevalence of 15% (95% CI: 13–17%) for *E. coli* in foods of animal origin, which is lower than the prevalence observed in the present study. The higher prevalence detected in reptiles may reflect differences in host ecology, environmental exposure, and microbial colonization dynamics between reptiles and food-producing animals. Overall, these findings demonstrate that *E. coli* is commonly present in reptilian populations globally and highlight the potential role of reptiles as reservoirs of enteric bacteria of public health importance.

*Salmonella* spp. are facultative anaerobic Gram-negative rod-shaped bacteria belonging to the Enterobacteriaceae family and are among the most significant zoonotic pathogens worldwide (Brown et al., 2021). Reptiles are well-recognized reservoirs of *Salmonella* (Mlangeni et al., 2024), and reptile-associated salmonellosis in humans has been documented for several decades (Bernar et al., 2023). In the current study, the pooled prevalence of *Salmonella* spp. was estimated at 33.3% (95% CI: 29.3–37.5%), indicating that approximately one-third of reptiles carried *Salmonella*. This prevalence was higher than that observed for *E. coli* (25.1%), further supporting the established role of reptiles as important reservoirs of *Salmonella* and a potential source of zoonotic transmission. Our findings were comparable to those reported by Muslin et al. (2025), who estimated an overall pooled prevalence of *Salmonella* spp. in reptiles of 30.4%. The similarity between these estimates provides further evidence that *Salmonella* carriage is common among reptilian hosts worldwide. The observed variation may be explained by differences in reptile taxa, geographical distribution, and detection techniques. The consistently high prevalence observed across numerous studies suggests that *Salmonella* carriage is widespread among reptiles globally, highlighting the importance of reptiles as reservoirs of this pathogen and their potential role in the epidemiology of human salmonellosis.

The majority of studies in this review focused on reptile microbiome and pathogen surveillance involve captive (45.9%) and wild-caught (35.9%) reptiles, illustrating the primary contexts in which this research has been conducted. Captive populations are typically more accessible for repeated sampling and health monitoring, while wild-caught individuals offer valuable insights into the natural microbial communities associated with free-ranging reptiles. However, it’s important to note that the microbial composition of reptiles is significantly influenced by their environmental conditions. Therefore, when interpreting microbiological findings, one must consider the differences between captive and wild populations.

Environmental variation plays a crucial role in shaping the bacterial communities found in reptiles. When kept in captivity, factors such as changes in their habitat, diet and husbandry practices can significantly disrupt their normal microbiota, leading to a higher risk of opportunistic bacterial colonization. Additionally, the stress of captivity coupled with frequent handling and transport- can weaken their immune systems, making reptiles more vulnerable to bacterial infections (Ferreira Junior et al., 2009). Ferreira Junior et al. (2009) also highlighted that semi-open housing systems, which provide improved air circulation, natural temperature gradients, and less frequent handling, tend to support reptile health more effectively than stricter environments. Consequently, it’s important to recognize that the bacterial profiles we observe in captive reptiles may not truly reflect those found in their wild counterparts. Understanding these differences can help improve the care and well-being of reptiles in captivity.

Diet plays a crucial role in determining the presence of bacteria in captive reptiles (Jiang et al., 2017). Research shows that reptiles fed feeder rodents carrying contaminants tend to shed higher amounts of *Salmonella* spp. (Vrbova et al., 2018). Since feeder mice and rats are known to be reservoirs for *Salmonella*, they can pose a risk for spreading bacteria within collections of captive reptiles (Chlebicz and Śliżewska, 2018). These findings highlight the importance of careful husbandry practices, especially when it comes to feeding routines, as they can greatly affect the levels of zoonotic bacteria found in these animals.

The large proportion of studies on captive reptiles may also reflect growing public health concerns associated with the increasing popularity of reptiles as companion animals. Historically, chelonians were considered the principal source of reptile-associated salmonellosis due to their widespread ownership as pets (Chiodini and Sundberg, 1981). However, the expanding domestication of snakes and lizards has shifted attention toward these taxa, which have consistently been reported to exhibit high rates of *Salmonella* carriage and shedding (Chen et al., 2010; Kikillus et al., 2011; Lukac et al., 2015). This concern is supported by evidence identifying lizards as an increasingly important source of reptile-associated salmonellosis in humans (Pees et al., 2013). The increased representation of captive reptiles in the literature is therefore likely driven not only by ease of sampling but also by the need to better understand zoonotic risks associated with the exotic pet trade.

This systematic review and meta-analysis revealed substantial variation in the methodologies used to detect and identify bacterial pathogens in reptiles. Nevertheless, conventional microbiological approaches remained the most widely used methods across the reviewed studies. The highest pooled prevalence of *Salmonella* spp. was observed in studies that combined culture with serotyping, yielding a prevalence of 48.1%. This was followed by studies using a combination of culture, biochemical characterisation, and serotyping, which produced a pooled prevalence of 35.5%. Similarly, it was observed that, in *E. coli*, the highest pooled prevalence was reported for studies that used a combination of culture, biochemical testing, and serotyping, at 50.3%. This was followed by culture with biochemical identification, which yielded a prevalence of 23.1%. This predominance likely reflects the long-established role of culture-based methods such as the diagnostic gold standard for bacterial isolation, particularly for enteric pathogens such as *Salmonella* spp. and *E. coli*. Culture methods are relatively inexpensive, widely accessible and allow the recovery of viable isolates for subsequent phenotypic characterisation, antimicrobial susceptibility tests, and epidemiological investigations (Andrews et al., 2018; Mooijman et al., 2019). The frequent incorporation of biochemical tests and serotyping further allows taxonomic confirmation and differentiation of clinically relevant serovars, which is particularly important for assessing zoonotic risks associated with reptile-associated bacteria (Grimont and Weill, 2007).

Wild snakes eat all kinds of live mammals and birds. Warm-blooded prey are natural carriers of subspecies I and host-generalist serovars such as *S. typhimurium* and *S*. Newport (Smith, 2025). Pet snakes, on the other hand, eat pet-shop mice and rats which are frozen, thawed, and biosecurity screened. Temperature abuse greatly reduces the opportunity for chronic exposure to a true warm-blooded reservoir. Rates of *Salmonella* carriage among snakes tend to be greater than in captivity populations because stress caused by captivity can cause immunodeficiency. This allows for an influx of certain cold-blooded adapted lineages (subspecies *diarizonae* [IIIb] and *salamae* [II]) to enter the intestines of captive snakes. These very adapted strains overpopulate and competitively exclude subspecies I strains due to faster replication rates and limited sites to bind to in the intestine, while wild snakes do not see this colonization due to stable gut microbiomes already in place. Increased carriage is not the only factor that makes *Salmonella* dangerous to those who own snakes. Clinical concern is also raised due to subspecies IIIa (*S. arizonae*), as this subspecies has been shown to cause cases of acute gastroenteritis and diarrhea in children who have had contact with pet reptiles (Abba et al., 2017). Children are particularly susceptible to systemic spread from *Salmonella* because their immune systems have not fully developed and they are more likely to perform hand-to-mouth contact after handling infected surfaces in the reptile’s enclosure.

*Salmonella bongori* had a complete absence of prevalence in all datasets pulled for this review. *S. bongori* colonizes reptiles as well but may have been limited due to both geographic endemicity and competition from other *Salmonella* species in captivity (Pees et al., 2023; Muslin et al., 2025). *S. bongori* do not have worldwide prevalence naturally and is only found prevalently in certain Mediterranean regions and areas of Southern Europe. Due to the fact that most of these samples came from either North America, Northern Europe, or tropical importing countries for exotic pets, the subject animal samples did not overlap with the natural habitat for *S. bongori*. It also faces competition with other *Salmonella* species that are more adapted to mammals in captivity. In pet shops and reptile breeding facilities, *S. enterica* strains dominate and outcompete *S. bongori* for binding sites within the intestines of these reptiles (Dróżdż et al., 2021).

Studies that relied on culture followed by biochemical identification alone reported a pooled prevalence of 26.9% for *Salmonella* spp. and 23.1% for *E. coli*. Although these methods remain valuable for routine diagnostics, their reliance on phenotypic characteristics may result in reduced specificity when distinguishing closely related bacterial taxa. Phenotypic variation among isolates and the potential for atypical biochemical profiles can contribute to misidentification (Janda and Abbott, 2007), particularly when dealing with diverse environmental and reptile-associated bacterial communities. Consequently, studies that are based solely on culture and biochemical characterisation may have underestimated the bacterial diversity or introduced inconsistencies in pathogen identification across investigations.

The integration of molecular techniques, particularly PCR, was comparatively limited with a pooled prevalence estimate of (22.1%) for *Salmonella* spp. and (14.9%) for *E. coli,* despite their recognized advantages. PCR-based assays offer improved sensitivity, specificity, and rapid detection of bacterial pathogens, including those present at low abundance or in samples containing competing microbial flora (Malorny et al., 2004). The use of culture followed by PCR in a subset of studies improved diagnostic accuracy by allowing the genetic confirmation of isolates and reducing the risk of false-positive identifications associated with phenotypic methods alone. However, the relatively low adoption of PCR-based approaches may reflect financial constraints, limited laboratory infrastructure, and the historical focus of many reptile microbiology studies conducted before the widespread availability of molecular diagnostics.

Advanced analytical approaches, MALDI-TOF MS and whole-genome sequencing (WGS), were infrequently reported in the included studies, with pooled prevalence estimates of 8.9 and 20.1%, respectively for *Salmonella* spp. and culture combined with MALDI-TOF MS at 3.9% for *E. coli*. Despite their transformative impact on contemporary microbial diagnostics. MALDI-TOF MS provides rapid, accurate, and cost-effective bacterial identification based on protein spectral profiles and has become an increasingly standard tool in clinical microbiology laboratories (Patel, 2015). Similarly, WGS offers unparalleled resolution for species identification, serovar determination, virulence profiling, antimicrobial resistance characterization, and phylogenetic analyses (Allard et al., 2013; Ashton et al., 2015). The very limited application of these technologies in reptile-associated bacterial research likely reflects their relatively recent emergence, higher operational costs, and the specialized bioinformatics expertise required for data analysis. Nevertheless, the growing accessibility of genomic technologies suggests that future studies can increasingly adopt these approaches, thus improve pathogen surveillance and advance our understanding of bacterial ecology in reptile hosts.

The results from the meta-analysis demonstrated substantial variation in the prevalence of *Salmonella* spp. among major reptile taxa, indicating that host ecology and biology may play important roles in shaping bacterial carriage patterns. Snakes exhibited the highest pooled prevalence of 49.4%. These findings are broadly consistent with those reported by Muslin et al. (2025), who found the highest prevalence in snakes (63.1%), followed by lizards (33.6%), turtles (11.2%), and crocodilians (10.5%). The similarity between the two studies, despite differences in study inclusion criteria and geographical coverage, strengthens the evidence that snakes harbor *Salmonella* consistently at higher rates than other reptilian groups.

The elevated prevalence observed in snakes may be attributable to several ecological and physiological factors. One widely accepted explanation relates to their predominantly carnivorous feeding behavior. Snakes frequently consume rodents, birds, amphibians, and other vertebrates that may serve as reservoirs of *Salmonella*, thereby facilitating bacterial acquisition through the food chain (Schröter et al., 2004; Pantuzza Ramos et al., 2019; Song et al., 2023). In addition, *Salmonella* has been shown to persist within the gastrointestinal tract of reptiles without causing overt disease, supporting the notion that many snake species function as asymptomatic carriers and natural reservoirs of the bacterium (Mermin et al., 2004; Hoelzer et al., 2011). Environmental exposure may also contribute to the higher prevalence observed in snakes. Many captive snakes are maintained in enclosed terrarium systems where repeated contact with contaminated substrates, water sources, and fecal material can facilitate bacterial transmission and reinfection (Schröter et al., 2004; Lukac et al., 2015). Furthermore, the intermittent shedding patterns exhibited by reptiles may lead to prolonged environmental contamination, increasing opportunities for transmission among individuals sharing the same enclosure (Woodward et al., 1997).

In contrast, Chelonians exhibited significantly less *Salmonella* prevalence than snakes and lizards. Several factors may explain this pattern. Seasonal fluctuations in feeding activity and metabolism, particularly among temperate species undergoing hibernation or brumation, have been associated with reduced rates of *Salmonella* isolation (Geue and Löschner, 2002; Cota et al., 2021). Differences in diet, habitat use, and gastrointestinal physiology may also influence colonization dynamics among chelonians. However, despite their comparatively lower prevalence, chelonians remain epidemiologically important because even low-level carriage can contribute to environmental contamination and zoonotic transmission, particularly in pet turtles, which have been repeatedly implicated in human salmonellosis outbreaks (Mermin et al., 2004; Bosch et al., 2016).

The results from the meta-analysis show that the prevalence of *E. coli* differed considerably among reptile taxa, with lizards exhibiting the highest pooled prevalence (36.8%), followed by crocodilians (25.3%), chelonians (21.9%), and snakes (16.8%). These findings suggest that host taxonomy may play an important role in shaping *E. coli* colonization patterns in reptiles. The comparatively high prevalence observed in lizards may be associated with their diverse diets, habitats, and gastrointestinal microbial communities. Lizards occupy a wide range of ecological niches and include herbivorous, omnivorous, and insectivorous species, each of which is exposed to different environmental microbial sources. Herbivorous and omnivorous reptiles generally possess more complex gut microbiota due to the microbial fermentation required for plant digestion, creating favorable conditions for facultative anaerobes such as *E. coli* (Kohl et al., 2017). In addition, many lizard species have frequent contact with soil, vegetation, and, in some cases, human-modified environments, all of which may increase opportunities for exposure to enteric bacteria. Previous research has shown that factors such as diet, habitat, and captivity can strongly influence the composition of reptilian gut microbiota, potentially contributing to the higher prevalence observed in this group (Kohl et al., 2017). Within the lizard subgroup, iguanas had the highest pooled prevalence (56.5%). This result is likely linked to their predominantly herbivorous feeding habits, which support diverse microbial communities involved in plant digestion and fermentation (Kohl et al., 2017). In contrast, geckos showed a much lower prevalence (15.1%). As most gecko species are insectivorous and possess relatively simple digestive systems, they may provide fewer opportunities for the establishment and persistence of *E. coli* populations.

Chelonians showed an overall intermediate prevalence, although substantial variation was observed among subgroups. Terrapins recorded the highest prevalence (41.1%), followed by tortoises (24.9%) and turtles (14.2%). The elevated prevalence in terrapins may partly reflect the ability of *E. coli* to persist both within hosts and in surrounding environments (Mlangeni et al., 2025). Differences among chelonian groups may be explained by habitat-associated exposure risks. As semi-aquatic reptiles, terrapins inhabit environments where fecal contamination can accumulate, potentially increasing exposure to enteric bacteria through contaminated water and sediments. Aquatic systems are well-recognized reservoirs of enteric microorganisms and may facilitate repeated exposure and recolonization. Interestingly, this pattern differs from findings reported for some other reptile-associated bacterial taxa, where aquatic and semi-aquatic species have sometimes shown lower carriage rates than terrestrial species (Gilbert et al., 2014; Di Ianni et al., 2015).

Snakes exhibited the lowest pooled prevalence estimate in the present analysis. However, this finding should be interpreted with caution because several individual studies have reported relatively high *E. coli* occurrence in snake populations, particularly those maintained in captivity. For example, Pantuzza Ramos et al. (2019) found higher isolation rates in snakes (51.6%) than in lizards and chelonians (both 44.4%), while Dec et al. (2022) also reported snakes as the reptile group most frequently carrying *E. coli*. The lower pooled estimate observed here may therefore be influenced by differences among studies, including variation in host species, geographic regions, sampling strategies, and husbandry conditions. The carnivorous feeding habits of snakes may further contribute to variability in bacterial carriage, as prey-associated microorganisms and prolonged periods without feeding are known to influence gut microbial composition.

Crocodilians had the second-highest pooled prevalence (25.3%), although this estimate was based on a relatively small number of studies. The ecology of crocodilians may favor exposure to enteric bacteria because they spend much of their lives in aquatic habitats that receive microbial inputs from wildlife, livestock, and human activities. Their opportunistic feeding behavior also increases contact with diverse microbial sources. Several studies have reported the presence of *E. coli* as part of the normal intestinal microbiota of crocodilians, supporting their role as potential reservoirs of environmental enteric bacteria. For example, *E. coli* was isolated from nine gharials and eight dwarf crocodiles examined by Lovely and Leslie (2008). Although often regarded as a commensal organism, *E. coli* can also act as an opportunistic pathogen in crocodilians. Foggin (1992) reported that 47 out of 409 bacterial infections (11.5%) in crocodiles were attributed to *E. coli*, including cases of septicaemia in the Nile crocodile. These findings highlight the dual role of *E. coli* as both a common intestinal inhabitant and a potential cause of disease under favorable conditions.

This systematic review and meta-analysis have several limitations. (a) The search strategy was restricted to peer-reviewed articles published in English, excluding reviews, theses, and preprints as per PRISMA guidelines; consequently, relevant studies in other languages or unpublished formats may have been missed. (b) The number of available studies varied considerably across countries and continents; some regions had no data or too few publications to calculate pooled prevalence, while others had an unusually high number of reports, potentially biasing overall estimates. (c) High heterogeneity was present across all analyses, which complicates the interpretation of publication bias tests (e.g., funnel plots, regression, Begg’s adjusted rank correlation test). These evaluation tools can produce misleading results when a meta-analysis includes fewer than ten studies or exhibits substantial heterogeneity, making it difficult to assess true publication bias. Therefore, readers should exercise caution when interpreting both the pooled analyses and subgroup findings. (d) One limitation of this systematic review could be the minimum sample size of studies included (>10 reptiles). Although this was decided upon *a priori* to reduce bias introduced through statistical anomalies in smaller sample sizes, this could also lead to selection bias by excluding rare reptile taxa or uncommon habitats that are difficult to sample at greater abundances.

## 5 Conclusion

This study is the first systematic review and meta-analysis to estimate the global prevalence of *Escherichia coli* and *Salmonella* spp. in reptiles. The overall pooled prevalence was 33.3% for *Salmonella* spp. and 25.1% for *E. coli*, demonstrating that reptiles frequently harbor these clinically relevant bacteria. Significant differences in prevalence were observed across reptile taxa; snakes exhibited the highest *Salmonella* spp. prevalence, whereas lizards showed the highest *E. coli* prevalence (36.8%). The presence of *E. coli* in reptiles, particularly in lizards, raises additional public health and environmental concerns, as reptiles may act as reservoirs for pathogenic or antimicrobial-resistant *E. coli* strains. Furthermore, several *Salmonella* serovars of public health relevance were commonly identified among reptiles, underscoring their substantial threat as a source of *Salmonella* transmission to humans. These robust findings provide a valuable reference for veterinarians, environmental scientists, and public health professionals, facilitating evidence-based risk assessments and guiding future surveillance efforts in reptile-associated infections.

## Data Availability

The original contributions presented in the study are included in the article/Supplementary material, further inquiries can be directed to the corresponding author.

## Footnotes

1
https://www.meta-analysis.com/
