# Supplementary material for: Global prevalence of Salmonella spp. and Escherichia coli in reptile taxa: a systematic review and meta-analysis
Source: Front Microbiol. 2026 Oct 28;17:1940090. doi: 10.3389/fmicb.2026.1940090 (PMC13752144; doi:10.3389/fmicb.2026.1940090)
Supplement: Supplementary file 1 [file Data_Sheet_1.docx]

**Global Pooled Prevalence of *Salmonella* spp. and *Escherichia coli* in Reptiles: A Systematic Review and Meta-Analysis**

Lungile N Mlangeni^1^, Cormac Price^2^, Jane Nkhebenyane^3^, Tsepo Ramatla^3*^, Che Weldon^1^

^1^ Unit for Environmental Sciences and Management, North-West University, Potchefstroom, 2531, South Africa.

^2^ School of Biological and Environmental Sciences, University College Dublin, Belfield, Dublin 4, Ireland.

^3^ Centre for Applied Food Safety and Biotechnology, Department of Life Sciences, Central University of Technology, 1 Park Road, Bloemfontein, 9300, South Africa.

*Corresponding author

Tsepo Ramatla, PhD

Tell: +27 51507 3911, Email: [tramatla@cut.ac.za](mailto:tramatla@cut.ac.za)

**Supplementary Table S1.** Checklist of items to include when reporting a systematic review or meta-analysis.

| Section/topic | # | Checklist item | Reported on page # |
| --- | --- | --- | --- |
| **TITLE** | | | |
| Title | 1 | Identify the report as a systematic review, meta-analysis, or both. | Title |
| **ABSTRACT** | | | |
| Structured summary | 2 | Provide a structured summary including, as applicable: background; objectives; data sources; study eligibility criteria, participants, and interventions; study appraisal and synthesis methods; results; limitations; conclusions and implications of key findings; systematic review registration number. | Abstract |
| **INTRODUCTION** | | | |
| Rationale | 3 | Describe the rationale for the review in the context of what is already known. | Introduction |
| Objectives | 4 | Provide an explicit statement of questions being addressed with reference to participants, interventions, comparisons, outcomes, and study design (PICOS). | Introduction |
| **METHODS** | | | |
| Protocol and registration | 5 | Indicate if a review protocol exists, if and where it can be accessed (e.g., Web address), and, if available, provide registration information including registration number. | Methods |
| Eligibility criteria | 6 | Specify study characteristics (e.g., PICOS, length of follow-up) and report characteristics (e.g., years considered, language, publication status) used as criteria for eligibility, giving rationale. | Materials and methods |
| Information sources | 7 | Describe all information sources (e.g., databases with dates of coverage, contact with study authors to identify additional studies) in the search and date last searched. | Materials and methods |
| Search | 8 | Present full electronic search strategy for at least one database, including any limits used, such that it could be repeated. | Materials and methods |
| Study selection | 9 | State the process for selecting studies (i.e., screening, eligibility, included in systematic review, and, if applicable, included in the meta-analysis). | Materials and methods |
| Data collection process | 10 | Describe method of data extraction from reports (e.g., piloted forms, independently, in duplicate) and any processes for obtaining and confirming data from investigators. | Materials and methods |
| Data items | 11 | List and define all variables for which data were sought (e.g., PICOS, funding sources) and any assumptions and simplifications made. | Materials and methods |
| Risk of bias in individual studies | 12 | Describe methods used for assessing risk of bias of individual studies (including specification of whether this was done at the study or outcome level), and how this information is to be used in any data synthesis. | Materials and methods |
| Summary measures | 13 | State the principal summary measures (e.g., risk ratio, difference in means). | Materials and methods |
| Synthesis of results | 14 | Describe the methods of handling data and combining results of studies, if done, including measures of consistency (e.g., I^2^) for each meta-analysis. | Materials and methods |
| Risk of bias across studies | 15 | Specify any assessment of risk of bias that may affect the cumulative evidence (e.g., publication bias, selective reporting within studies). | Materials and methods |
| Additional analyses | 16 | Describe methods of additional analyses (e.g., sensitivity or subgroup analyses, meta-regression), if done, indicating which were pre-specified. |  |
| RESULTS | | | |
| Study selection | 17 | Give numbers of studies screened, assessed for eligibility, and included in the review, with reasons for exclusions at each stage, ideally with a flow diagram. | Results |
| Study characteristics | 18 | For each study, present characteristics for which data were extracted (e.g., study size, PICOS, follow-up period) and provide the citations. | Results |
| Risk of bias within studies | 19 | Present data on risk of bias of each study and, if available, any outcome-level assessment (see Item 12). | Results |
| Results of individual studies | 20 | For all outcomes considered (benefits or harms), present, for each study: (a) simple summary data for each intervention group and (b) effect estimates and confidence intervals, ideally with a forest plot. | Results |
| Synthesis of results | 21 | Present results of each meta-analysis done, including confidence intervals and measures of consistency. | Results |
| Risk of bias across studies | 22 | Present results of any assessment of risk of bias across studies (see Item 15). | Results |
| Additional analysis | 23 | Give results of additional analyses, if done (e.g., sensitivity or subgroup analyses, meta-regression) (see Item 16). | Results |
| DISCUSSION | | | |
| Summary of evidence | 24 | Summarize the main findings including the strength of evidence for each main outcome; consider their relevance to key groups (e.g., health care providers, users, and policy makers). | Discussion |
| Limitations | 25 | Discuss limitations at study and outcome level (e.g., risk of bias), and at review level (e.g., incomplete retrieval of identified research, reporting bias). | Limitations |
| Conclusions | 26 | Provide a general interpretation of the results in the context of other evidence, and implications for future research. | Conclusions |
| FUNDING | | | |
| Funding | 27 | Describe sources of funding for the systematic review and other support (e.g., supply of data); role of funders for the systematic review. |  |

**Supplementary Table S2.** Search strains in databases.

**PubMed** (*n* = 303)

(Enterobacteriaceae OR Enterobacteria OR "enteric bacteria" OR Salmonella OR "Escherichia coli" OR E. coli OR Klebsiella OR Citrobacter OR Enterobacter OR Proteus OR Serratia OR Morganella OR Edwardsiella OR Yersinia OR Hafnia OR Pantoea OR Providencia) AND (Reptile* OR Reptilia OR Snake* OR Serpentes OR Lizard* OR Lacertilia OR Turtle* OR Tortoise* OR Testudines OR Crocodil* OR Alligator* OR Caiman* OR Terrapin*)

**ScienceDirect** (*n* =370)

(Enterobacteriaceae OR "enteric bacteria" OR Salmonella OR "Escherichia coli" OR Klebsiella OR Citrobacter) AND (Reptile OR Reptilia OR Snake OR Lizard OR Turtle OR Tortoise OR Crocodilian)

**Scopus** (*n* = 440)

(Enterobacteriaceae OR Enterobacteria OR "enteric bacteria" OR Salmonella OR "Escherichia coli" OR E. coli OR Klebsiella OR Citrobacter OR Enterobacter OR Proteus OR Serratia OR Morganella OR Edwardsiella OR Yersinia OR Hafnia OR Pantoea OR Providencia) AND (Reptile* OR Reptilia OR Snake* OR Serpentes OR Lizard* OR Lacertilia OR Turtle* OR Tortoise* OR Testudines OR Crocodil* OR Alligator* OR Caiman* OR Terrapin*)

**Google Scholar** (*n* = 997)

Enterobacteriaceae OR Salmonella OR "E. coli" OR Klebsiella reptile OR reptilia OR snake OR lizard OR turtle OR "testudines"

**MEDLINE** (*n* = 14)

(Enterobacteriaceae [MeSH Terms] OR Enterobacteriaceae[tiab] OR Salmonella[tiab] OR "Escherichia coli"[tiab] OR Klebsiella[tiab]) AND (Reptiles [MeSH Terms] OR Reptile*[tiab] OR Snake*[tiab] OR Lizard*[tiab] OR Turtle*[tiab] OR Crocodil*[tiab])

**Supplementary Table S3.** Characteristics of the reviewed articles included in the meta-analysis.

| **Column1** | **Authors & years** | **Country** | **Source** | **Sample** | **Total** | ***Salmonella* spp.** | ***E. coli*** |
| --- | --- | --- | --- | --- | --- | --- | --- |
| 1 | Gopee, et al., 2000 | Trinidad | Captive reptiles | Cloacal/fecal samples | 173 | – | 95 |
| 2 | Goupil et al., 2012 | USA | Captive snakes | Cloacal/fecal samples | 12 | 11 | – |
| 3 | Fitzgerald et al., 2013 | USA | Snakes | Cloacal/fecal samples | 31 | 8 | 8 |
| 4 | Jho et al., 2011 | Korea | Snakes | Oral cavity swabs and cloacal swabs | 20 | 2 | 14 |
| 5 | Pereira et al., 2017 | Brazil | Snakes | Oral cavity | 12 | 1 | 3 |
| 6 | Santoro et al., 2006 | USA | Green turtles (Chelonia mydas) | Cloacal and body/ surface | 70 | – | 6 |
| 7 | Plows et al., 1968 | USA | Terrapins | Stool | 12 | 1 | – |
| 8 | Schröter et al., 2006 | Germany | Snakes | Fecal, cloacal | 20 | 13 | – |
| 9 | Galgano et al., 2023 | Italy | Snakes | Cloacal/fecal samples from reptiles | 10 | 8 | – |
| 10 | Centeno et al., 2020 | Guatemala | Turtles | Cloacal | 63 | 3 | – |
| 11 | Muñoz-Ibarra et al., 2022 | Peninsula | Trachemys scripta (red-eared slider turtle) and Python regius (royal python). | Faecal/cloacal/oral samples | 345 | – | 15 |
| 12 | Trotta et al., 2021 | Italy | Loggerhead Sea Turtles (Caretta caretta) | Cloacal and clinical samples | 98 | 4 | 4 |
| 13 | Guyomard-Rabenirina et al., 2020 | Guadeloupe | Iguanas | Faecal samples | 46 | – | 1 |
| 14 | Marin et al., 2022 | Spain | Tortoise, turtles, terrapins | Cloacal/faecal samples | 152 tortoises (n = 81), pond turtles (n = 37) and sea turtles (n = 34) | 29 | – |
| 15 | Sharina et al., 2023 | Malaysia | Python (snake) | Oral lesions/swabs | 40 | 3 | 4 |
| 16 | Hallinger et al., 2019 | Germany | Lizards | Faecal samples | 748 | 249 | – |
| 17 | Bautista-Trujillo et al., 2020 | Mexico | Iguanas | Cloacal swabs | 240 | – | 149 |
| 18 | Wheeler et al., 2012 | Ecuador | Marine and land iguanas and Galapagos tortoises | Cloacal/faecal samples | 18 (marine 12, land 6) tortoise 10 | – | giant tortoises (n=10), land iguanas (n=29), and rmarine iguanas (n=50) |
| 19 | Franco et al., 2011 | Ecuador | Iguanas | Cloacal/faecal samples | 63 | 62 | – |
| 20 | Romero et al., 2015 | Czech Republic | Iguanas | Choanal and cloacal swabs | 20 | 9 | – |
| 21 | Schmidt et al., 2014 | Germany | Snakes and legless lizard | Cloacal swabs | 56 | 8 | 35 |
| 22 | Pawlak et al., 2020 | Poland | Snakes | Cloacal swabs | 45 | 10 | – |
| 23 | Bemis et al., 2007 | USA | Snakes | Cloacal/faecal samples | 77 | 77 | – |
| 24 | Kruse et al., 2015 | USA | Tortoise | Faecal samples (fresh vs frozen) | 10 | 10 | – |
| 25 | Di Ianni et al., 2015 | Italy | Turtles and tortoises | Conjunctival swabs | 34 | 1 | – |
| 26 | Ajayi et al., 2015 | Nigeria | Lizards | Oral and cloacal swabs | 183 | 21 | 41 |
| 27 | Song et al., 2023 | China | Snakes, lizards, chelonians | Cloacal/faecal samples | 177 (104 from snakes, 21 from lizards, and 52 from chelonians) | 46 (snakes n= 32, lizards n= 2 and chelonians n=4) | – |
| 28 | Lecis et al., 2011 | Italy | Tortoise | Cloacal swabs | 30 | 3 | – |
| 29 | Cortes-Cortes et al., 2016 | Mexico | Turtles | Cloacal/faecal samples | 71 | – | 11 |
| 30 | Heynol et al., 2015 | Germany | Turtle | Oral swabs | 20 | 16 | – |
| 31 | Ives et al., 2017 | USA | Turtle | Cloacal swabs + sand/water samples | 32 (9 Leatherback sea turtles, 14 Hawksbill sea turtles, 9 Green sea turtles) | 4 | – |
| 32 | da Silva Argôlo et al., 2013 | Brazil | Snakes | Cloacal/faecal samples | 31 | 18 | – |
| 33 | Fasaei & Tamai, 2017 | Iran | Lizards, terrapin, snake | Faecal samples | 17 (lizard 3, terrapin 9, snake 5) | 10 (lizard 2, terrapin 6, snake 2) | – |
| 34 | Marenzoni et al., 2022 | Italy | Tortoise | Cloacal/oral swabs | 180 turtles (121 swabs) | 45 | – |
| 35 | Hacioglu & Tosunoglu, 2014 | Turkey | Terrapin, snake | Cloacal/fecal samples + aquatic isolates | 40 (35 terrapin, 5 snake) |  | 12 (terrapin) |
| 36 | Somanath, 2022 | India | Snake | Cloacal/faecal samples | 30 | 20 | – |
| 37 | Weerakhun et al., 2022 | Thailand | Snakes, lizards, turtles, crocodiles | Cloacal/faecal samples | 257 (turtles 146, snakes 77, lizards 33, crocodile 1) | 93/257 (snakes 54, lizards 19, turtles 20). | – |
| 38 | Ofori et al., 2023 | Ghana | Lizard | Gastrointestinal contents/faecal | 37 | 6 | 32 |
| 39 | Ogbodogbo et al., 2021 | Nigeria | Gekho | Intestinal samples | 51 | 43 | – |
| 40 | Wang et al., 2024 | China | Turtle | Cloacal/faecal + clinical samples | 480 | 46 | – |
| 41 | Junior et al., 2015 | Brazil | Turtle | Cloacal/faecal samples | 116 | 93 | – |
| 42 | Lee et al., 2024 | Australia | Lizard | Faecal samples (longitudinal) | 87 | 87 | 83 |
| 43 | Morais et al., 2011 | Brazil | Turtles | Oral and cloacal swabs | 48 | 2 | 14 |
| 44 | Mathewson, 1979 | USA | Lizards | Intestinal/cloacal samples | 67 | 33 | 13 |
| 45 | Chiacchio et al., 2014 | Brazil | Terrapin | Cloacal samples | 39 |  | 5 |
| 46 | Wang et al., 2020 | China | Turtle | Fecal | 290 | 42 |  |
| 47 | Valença et al., 2025 | Portugal | Bearded dragon, gekho | Cloacal/faecal samples | 78 (20 bearded dragon, 58 gekho) | – | 1 |
| 48 | Umbraško et al., 2023 | Europe | Turtles | Cloacal/faecal samples | 16 | 11 | – |
| 49 | Gruszynski et al., 2014 | USA | Turtles, snake | Faecal | 32 (31 turtle, 1 snake) | 7 (6 turtle, 1 snake) | – |
| 50 | Ahasan et al., 2017 | Australia | Turtle | Cloacal/faecal samples | 73 | – | 9 |
| 51 | Eibach et al., 2019 | Ghana | Gekho | Cloacal/faecal samples | 30 | – | 4 |
| 52 | Benhafid et al., 2025 | Algeria | Turtle | Cloacal/faecal samples | 24 | 13 | 10 |
| 53 | Marin et al., 2013 | Spain | Turtle | Cloacal/faecal samples | 200 | 11 |  |
| 54 | Pace et al., 2019 | Italy | Turtle | Gastrointestinal/cloacal samples | 30 | – | 2 |
| 55 | Galosi et al., 2021 | South Africa | Tortoise | Cloacal/faecal samples | 10 | 9 | 1 |
| 56 | Maciel et al., 2010 | Brazil | Lizard (tegus) | Cloacal/faecal samples | 30 | 26 | – |
| 57 | Gong et al., 2014 | China | Turtles | Cloacal/faecal samples | 41 | 16 | – |
| 58 | Holgersson et al., 2016 | USA | Turtle eggs | Eggshell + hatchling samples | 52 | 18 | – |
| 59 | Taiwo et al., 2021 | Nigeria | Gekho | Cloacal/faecal samples | 138 | 14 | – |
| 60 | Ramos et al., 2019 | Brazil | Lizards, chelonians, snakes | Cloacal/faecal samples | 76 (15 lizards, 16 chelonians, and 45 snakes) | 26 | 52 |
| 61 | Adhikari et al., 2025 | Nepal | Crocodile | Cloacal swabs | 28 | 12 | 13 |
| 62 | McWhorter et al., 2021 | Australia | Turtle, snake, lizard (wild caught), and turtle, tortoise, lizard, snake, crocodile (zoo species) | Cloacal/faecal samples | 130 (5 turtles, 14 snakes, 111 lizards) (wild caught) and 122 (zoo) | 52 (1 turtle, 7 snakes and 44 lizards) and 42 (zoo) | – |
| 63 | Nwachukwu et al., 2014 | Nigeria | Gekho | Faecal droppings | 500 | 129 | 62 |
| 64 | Bindaco et al., 2020 | Brazil | Turtle | Oesophageal samples | 42 (33 alive, 9 dead) | – | 8 |
| 65 | Abba et al., 2017 | Malaysia | Snakes | Oral and cloacal swabs | 27 | 1 | 3 |
| 66 | Oludairo, 2017 | Nigeria |  | Faecal samples | 12 |  | 2 |
| 67 | Burdick et al., 1984 | USA | Lizards | Cloacal/faecal samples | 100 | 33 | – |
| 68 | Ríos et al., 2022 | USA | Iguanas | Meat samples | 13 | 8 | – |
| 69 | Uhart et al., 2011 | Argentina | Crocodile | Cloacal/faecal samples | 102 | 79 (peak) | – |
| 70 | Corrente et al., 2004 | Italy | Snakes, tortoises, chameleons, lizards | Faecal samples | 91 | 46 | – |
| 71 | Kourany &Telford, 1981 | Panama | Lizard | Cloacal/faecal samples | 447 | 131 | – |
| 72 | Pace et al., 2019 | Italy | Turtle | Cloacal/faecal samples | 35 | – | 5 |
| 73 | Ruzhanova-Gospodinova et al., 2024 | Bulgaria | Snakes | Laryngeal and oral swabs | 29 | – | 1 |
| 74 | Marenzoni et al., 2015 | Italy | Tortoises, turtles | Cloacal/faecal samples | 78 (38 tortoises, 40 turtles) | Tortoises 3 and turtles 15 | – |
| 75 | Campagner et al., 2012 | Brazil | Snakes | Cloacal/faecal samples | 224 | 4 | 13 |
| 76 | Wang et al., 2024 | China | Crocodile | Food/environmental samples | 34 | – | 16 |
| 77 | Molina-López et al., 2015 | Spain | Terrapin | Cloacal/fecal samples | 44 | 2 | – |
| 78 | Bošnjak et al., 2016 | Serbia | Snakes, lizards and turtles | Cloacal swabs | 88 (34 snakes, 47 lizards and 7 turtles) | 19- (5 snakes, 10 lizards and 4 turtles) | – |
| 79 | Mengistu et al., 2022 | Spain | Terrapin | Cloacal/fecal samples | 91 | 4 | 17 |
| 80 | Moreno et al., 1995 | Spain | Lizard, turtle | Cloacal/fecal samples | 55 (34 turtles, 21 lizards) | Turtles n=6, Lizards n=19 | – |
| 81 | Mlangeni et al., 2024 | South Africa | Chameleons, lizards, snakes, tortoises | Cloacal/fecal samples | 19 (lizards (lizards (n=6), snakes (n=3), chameleons (n=7), and tortoises (n=3) | 30 isolates: chameleons (3 isolates), lizards (2 isolates), snakes (3 isolates), and tortoises (2 isolates) | – |
| 82 | Santana-Hernández et al., 2023 | Spain | Snake | Cloacal samples | 73 | 15 | – |
| 83 | Ilayaraja et al., 2024 | India | Snake | Oral & cloacal swabs | 10 | – | 1 |
| 84 | Yak et al., 2015 | Singapore | Snake | Oral swabs | 10 | 1 | 1 |
| 85 | Pereira et al., 2017 | Brazil | Snake | Oral swabs | 42 (30 healthy and 12 sick) | 1 (sick) | 3 (sick), 8 (healthy) |
| 86 | Lazarkevich et al., 2024 | Bulgaria | Lizard, tortoise | Cloacal samples | 110 (86 lizards, 24 tortoises) | Tortoise 18 | Lizards 4 |
| 87 | Bertolini et al., 2021 | Brazil | Snakes | Tissue samples & oral/cloacal swabs | 10 | 6 | – |
| 88 | Cristina et al., 2022 | Romania | Chelonians, snakes, lizards | Cloacal swabs/fecal samples | 389 (166 chelonians, 98 snakes, 125 lizards) | 18 | 27 |
| 89 | Pfleger et al., 2003 | Austria | Turle, snake, lizard, chameleon | Cloacal/fecal samples | 103 | Snakes (25), lizards"saurians"(18), turtles (3) | – |
| 90 | Dec et al., 2022 | Poland | Snakes, lizards, turtles, crocodiles | Cloacal/fecal samples | 67 (snakes n=31, lizards n=18, turtles n=18) | 48 (snakes 27, lizards 14 and turtles 7) | – |
| 91 | Schröter et al., 2004 | Germany | Snakes | Cloacal swabs | 16 | 13 | – |
| 92 | Shnaiderman-Torban et al., 2019 | Israel |  | Fecal samples | 47 | – | 7 reptile species |
| 93 | Unger et al., 2024 | Germany | Lizards, snakes, turtles, tortoises | Cloacal/fecal samples | 184 samples- 60 sample batches and 92 shipments | 69 | 116 |
| 94 | Kurnianto et al., 2019 | Indonesia | Snakes, lizards, tortoises | Fecal samples | 129 (72 snakes, 43 lizards, 14 tortoises) | 140 | 125 |
| 95 | Jorge et al., 2023 | Mexico | Turtle | Cloacal swabs | 47 | – | 100 |
| 96 | Scheelings, 2008 | Australia | Turtle, lizards, snakes, monitor | Cloacal/fecal samples | 71 (turtles 20, monitor 15 (skink25) (snake 11) | Snakes 9 | – |
| 97 | Seepersadsingh & Adesiyun, 2003 | Trinidad |  | Cloacal/fecal samples | 14 | 2 | – |
| 98 | Doden et al., 2021 | USA | Turtle | Cloacal/fecal samples | 341 | 9 | – |
| 99 | Abatcha et al., 2013 | Malaysia | Snakes | Cloacal swabs | 42 | 16 | – |
| 100 | Chen et al., 2010 | Taiwan | Snakes, lizards, turtles | Cloacal/fecal samples | 476 (snakes-33, lizards-43, turtles-400) | Snakes 23, lizards 27, and turtles 97 | – |
| 101 | Sumiyama et al., 2020 | Japan | Lizard | Cloacal/fecal samples | 79 | 24 | – |
| 102 | Tomastikova et al., 2017 | Czech Republic | Lizards, chelonians, snakes | Cloacal/fecal samples | 211 | Lizards 2, snakes 3 and chelonians 4 | – |
| 103 | Valley & Abatcha, 2014 | Malaysia | Snakes | Cloacal/fecal samples | 42 | 16 | – |
| 104 | Madsen, 1996 | Zimbabwe | Crocodile meat | Meat samples (fresh/frozen) | 160 (20 fresh meat, 140 frozen meat) | 6 samples of fresh meat, and from 28 samples of frozen meat | – |
| 105 | Liu et al., 2013 | USA | Terrapin | Cloacal/fecal samples | 29 | 20 | 142 |
| 106 | Scott, 1996 | USA | Alligator | Cloacal/fecal samples, tissue samples | 100 | 20 farmed, 2 wild | – |
| 107 | Burnham et al., 1998 | USA | Iguana | Fecal samples | 12 | 7 | – |
| 108 | Bastos et al., 2008 | Brazil | Snake | Oral/cloacal swabs | 17 | 5 | 2 |
| 109 | Adesiyun et al., 1998 | Spain | Snakes, tortoises, iguanas | Cloacal/fecal samples | 61 (35 snakes "squamates",20 tortoises,6 iguana) | Snakes-17, Tortoises 1 | – |
| 110 | Kuroki et al., 2019 | Japan | Terrapin | Cloacal/fecal samples | 227 | 189 | – |
| 111 | Lukacet al., 2015 | Croatia | Lizards, chelonians, snakes | Cloacal/fecal samples | 200 (31 lizards, 79 chelonians,90 snakes) | 15 lizards, 8 snakes, 3 turtles | – |
| 112 | Sumiyama et al., 2020 | Japan | Lizards | Cloacal/fecal samples | 709 | 15 | – |
| 113 | Kuroki et al., 2013 | Japan | Snakes | Cloacal/fecal samples | 87 | 51 | – |
| 114 | Bjelland et al., 2020 | Norway | Snakes, lizards, chelonians | Cloacal/fecal samples | 103 (53 snakes, 15 lizards, 35 chelonians) | 44 (33 snakes, 10 lizards, 1 chelonians) | – |
| 115 | Jegede et al., 2016 | Nigeria | Snake | Cloacal/fecal samples | 30 | 30 | – |
| 116 | Nakadai et al., 2005 | Japan | Lizards, turtles, snakes | Cloacal/fecal samples | 112 (18 turtles, 71 lizards, 23 snakes) | 83 (23 snakes, 13 turtles, and 47 lizards) | – |
| 117 | Back et al., 2016 | Korea | Turtles | Cloacal/fecal samples + environmental swabs | 34 | 17 | – |
| 118 | Sharma et al., 2020 | Grenada, West Indies | Tortoise | Cloacal/fecal samples | 114 | 15 | – |
| 119 | Baling & Mitchell, 2021 | New Zealand | Lizards | Cloacal/fecal samples | 221 | 12 | – |
| 120 | Kikillus et al., 2011 | New Zealand | Lizards, turtles | Cloacal/fecal samples | 378 (263 lizards, 115 turtles) | 43 | – |
| 121 | Moroni et al., 2025 | Italy | Terrapins | Cloacal/fecal samples | 83 | 10 | – |
| 122 | Mairiga et al., 2024 | Nigeria | Lizards | Cloacal/fecal samples | 70 | 24 | – |
| 123 | Sylvester et al., 2014 | Grenada, West Indies | Iguana | Cloacal/fecal samples | 62 | 34 | – |
| 124 | Münch et al., 2012 | United Arab Emirates | Lizards, snakes, turtles | Cloacal/fecal samples | 176 | 63 | – |
| 125 | De Silva et al., 2017 | Korea | Turtles | Cloacal/fecal samples | 35 | 21 | – |
| 126 | Madsen et al., 1998 | Zimbabwe | Crocodiles | Cloacal swabs | 67 | 18 | – |
| 127 | Middleton et al., 2014 | New Zealand | Lizards, tuatara | Fecal samples | Lizards 275 tuatara 620 | 18 lizards | – |
| 128 | Guyomard-Rabenirina et al., 2019 | Guadeloupe | Anoles, guanas and geckos | Cloacal/fecal samples | 426 (322 anoles, 69 iguanas and 35 geckos) | 64 (anoles 36 and iguanas 28) | – |
| 129 | Gopee et al., 2000 | Trinidad | Snakes, tortoises | Cloacal/fecal samples | 173 | 25 | – |
| 130 | Corrente et al., 2017 | Italy | Snakes, lizards, turtles | Cloacal/fecal samples | 100 (48 snakes, 42 lizards, 10 turtles | 26 snakes, 28 lizards, 3 tortoises | – |
| 131 | Iveson et al., 1969 | Western Australia | Lizards, snakes, tortoises, crocodles | Cloacal/fecal samples | 116 (70 lizards, 40 snakes, 4 tortoises, 2 crocodiles) | 97 (lizards n=54, snakes n=37, tortoise n=4, crocodile n=2) | – |
| 132 | Shane et al., 1990 | USA | Turtle | Cloacal/fecal samples | 197 | 24 | – |
| 133 | Abrahão et al., 2020 | Brazil | Lizard | Cloacal/fecal samples | 153 | 67 | – |
| 134 | Geue & Löschner, 2002 | Germany, Australia | Turtles, snakes, lizards | Cloacal/fecal samples | 159 | 86 | – |
| 135 | Sánchez-Jiménez et al., 2011 | Colombia | Turtles | Cloacal/fecal samples | 110 | 30 | – |
| 136 | Fieldsend et al., 2023 | USA | Gekho | Cloacal/fecal samples | 37 | 5 | – |
| 137 | Dutton et al., 2013 | West Indies | Turtles | Cloacal/fecal samples | 21 | 3 | – |
| 138 | Lockhart et al., 2008 | USA | Tortoises | Cloacal/fecal samples | 80 | 4 | – |
| 139 | Sá & Solari, 2001 | Brazil | Snakes, lizards, chelonians | Cloacal/fecal samples | 97(15 snakes, 24 lizards, 58 chelonians) | 38 (15 lizards, 8 snakes, 15 chelonians) | – |
| 140 | Manolis et al., 1991 | Australia | Crocodile | Cloacal/fecal samples | 336 | 93 | – |
| 141 | Zając et al., 2021 | Poland | Snakes, lizards, turtles, crocodiles | Cloacal/fecal samples + environmental swabs | 696 (358 snakes, 276 lizards, 60 chelonians, 2 crocodiles) | 597 (330 snakes, 99 lizards, 36 turtles) | – |
| 142 | Hidalgo-Vila et al., 2007 | Spain | Tortoise, turtles | Cloacal/fecal samples | 92 (Testudo graeca (Tortoise) (n = 16), (turtles) Emys orbicularis (n = 26) and Mauremys leprosa (n = 50) | Tortoise n=16, n=6 in M. leprosa and n=4 in E. orbicularis | – |
| 143 | Hidalgo-Vila et al., 2008 | Spain | Turtles | Cloacal/fecal samples | 211 (78 native and 94 exotic turtles from natural ponds and 39 exotic pet turtles) | n=5 native and n=91exotic turtles from natural ponds and n=2 exotic pet turtle | – |
| 144 | Clancy et al., 2016 | USA | Crocodile, chelonians, lizards, snakes | Cloacal/fecal samples | 175 (4 crocodilian, 23 chelonian, 57 lizard, and 91 snake) | 117 fecal, 20 cloacal | – |
| 145 | Sumiyama et al., 2014 | Japan | Lizards | Cloacal/fecal samples | 141 | 46 | – |
| 146 | Bertelloni et al., 2016 | Italy | Chelonians, snakes, lizards | Cloacal/faecal samples | 213 (62 chelonians, 135 lizards, 16 snakes) | n=29 chelonians, n =14 lizards, and n=1 snakes | – |
| 147 | Percipalle et al., 2011 | Italy | Tortoise | Cloacal/faecal samples | 287 (220 spur-thighed tortoises) (67 spur-thighed tortoises) | Spur-thighed tortoises 81 and 17 spur-thighed tortoises | – |
| 148 | Sting et al., 2013 | Germany | Lizards, snakes, chelonians | Cloacal/faecal samples | 539 (235 lizards, 193 snakes and 111 chelonians) | n=83 lizards, n=91 snakes and n=13 chelonians | – |
| 149 | Cummings et al., 2021 | USA | Turtle | Faecal/tissue samples | 17 | 1 | – |
| 150 | Wikström et al., 2014 | Sweden | Snakes, lizards, turtle | Cloacal/faecal | 63 (25 snakes, 37 lizards, 1 turtle) | 31 | – |
| 151 | Smith et al., 2012 | USA | Gekho | Cloacal/faecal samples | 110 | 88 | – |
| 152 | Jiménez et al., 2015 | Costa Rica | Gekho | Cloacal/faecal samples | 115 | n=5 | – |
| 153 | Gay et al., 2014 | France | Lizards, snakes, turtles, iguanas, crocodiles | Cloacal samples | 151 (38 lizards, 37 snakes, 32 turtles, 23 green iguanas and 21 caimans "crocodile") | n=35 (crocodiles 3, turtles 6, iguanas 6, lizards 8, snakes 12) | – |
| 154 | Russo et al., 2018 | Italy | Gekho | Cloacal/faecal samples | 70 | 24 | – |
| 155 | Prapasarakul et al., 2012 | Thailand | Snakes | Cloacal/faecal + environmental samples | 128 | 128 | – |
| 156 | Rush et al., 2020 | Grenada | Snakes | Cloacal/faecal samples | 45 | 16 | – |
| 157 | Chambers & Hulse, 2006 | USA | Turtles, snakes, lizards | Cloacal/faecal samples | 64 (10 turtles, 54 snakes) | 61 | – |
| 158 | Martínez et al., 2011 | Spain | Lizards | Cloacal/faecal samples | 20 | 6 | – |
| 159 | Scott & Foster, 1997. | USA | Alligators | Cloacal/faecal samples | 100 (71 free-ranging and 29 farmed) | 6 farmed, n=2 wild | – |
| 160 | Lankau et al., 2012 | Galápagos | Iguanas | Cloacal/faecal samples | 79 (50 marine, 29 land) | 47 | – |
| 161 | Grupka et al., 2006 | USA | Snakes | Cloacal/faecal samples | 41 | 41 | – |
| 162 | Callaway et al., 2011 | Australia | Gekho | Cloacal/faecal samples | 100 | 7 | – |
| 163 | Cambre et al., 1980 | USA | Snakes, lizards, turtles | Cloacal/faecal samples | 317 (snakes 125, lizards 129, turtles 63) | 117 (snakes 69, lizards 46, turtles 2) | – |
| 164 | Bae et al., 2025 | South Korea | Gekho | Cloacal/faecal samples | 76 | 33 out 107 isolates | – |
| 165 | Merkevičienė et al., 2022 | Lithuania | Lizards, snakes, turtles | Cloacal/faecal samples | 97 (69 snakes, 26 lizrds, 2 turtles) | 50 | – |
| 166 | Zhao et al., 2024 | China | Turtles, lizards, snake | Cloacal/faecal samples | 109- 87 turtles, 21 lizards, 1 snake | 71 from turtles, 14 from lizards | – |
| 167 | Pedersen et al., 2009 | Denmark | Snakes, turtles, lizards, crocodile | Cloacal/fecal samples | 59- (26 snakes, 10 turtles, 22 lizards, 1 crocodile) | 60 | – |
| 168 | Oros et al., 1998 | Spain | Iguana | Cloacal/fecal samples | 10 | 1 iguana was tested and was + | – |
| 169 | Parsons et al., 2015 | Australia | Lizards | Cloacal/fecal samples | In 2001, 179 cloacal samples from 51 individuals, in 2002, 151 cloacal samples were collected from 38 individuals, in 2006, 372 cloacal samples were collected from 29 sleepy lizards. | In 2001 86 isolates, 2002 68 isolates, and 2006 268 isolates | – |
| 170 | Prabhudev et al., 2018 | South India | Tortoise | Cloacal/fecal samples | 40 | – | 11 |
| 171 | Dipineto et al., 2012 | Italy | Tortoise | Cloacal/fecal samples | 53 | 26 | – |
| 172 | Dégi et al., 2023 | Romania | Snakes, lizards, chelonians | Cloacal/fecal samples | 67 | 29 (snakes 7, Lizards 19, chelonians 3) | – |
| 173 | Gordon & Cowling, 2003 | Australia | Crocodiles, turtles, snakes, lizards | Cloacal/fecal samples | 299 | – | Crocodiles 98, turtles 12, snakes 6, lizards 30 |
| 174 | Pulford et al., 2019 | Togo, Nigeria, Cameroon, Egypt, Tanzania, Kenya, South Africa, and Uganda. | Snakes | Cloacal/fecal samples | 106 | 97 | – |
| 175 | Dehghani et al., 2016 | Iran | Snakes | Oral cavity swabs | 11 | 2 | 1 |
| 176 | Nguyen et al., 2018 | Vietnam | Gekho | Cloacal/fecal samples | 101 | 24 | – |
| 177 | Otis & Behler, 1973 | USA | Turtle | Cloacal/fecal samples | 127 | 37 | – |
| 178 | Książczyk et al., 2021 | Poland | Snakes, turtles, lizards | Cloacal/fecal samples | 120 | – | 24 strains |
| 179 | Romero et al., 2016 | Slovenia | Snakes, lizards, chelonians | Cloacal/fecal samples | 74 (22 lizards, 44 snakes, 8 chelonians) | n=22 (snakes 17, lizards 4, chelonians 1) | – |
| 180 | Middleton, 2008 | New Zealand | Lizards | Cloacal/fecal samples | 703 | 33 | – |
| 181 | Rose, 2021 | New Zealand | Lizards | Cloacal/fecal samples | 176 | 4 | – |
| 182 | Thaller et al., 2010 | Ecuador | Iguana | Cloacal/fecal samples | 96 | – | 68 |
| 183 | Mlangeni et al., 2025 | South Africa | Terrapins, tortoises | Cloacal/fecal samples | 36 | – | 23 |
| 184 | Casey, 2011 | Indonesia | Gekho | Cloacal/fecal samples | 186 | 198 | 7 |
| 185 | Pawlak et al., 2024 | Poland | Snakes | Cloacal/fecal samples | 69 (59 n. Natrix and 10 n. Fasciata) | 27 N. natrix and 3 N. fasciata | – |
| 186 | Dec et al., 2022 | Poland | Snakes, turtles, lizards | Cloacal/fecal samples | 67 (31 snakes, 18 turtles, 18 lizards) | – | Snakes 16, turtles 8 and lizards 8. |
| 187 | Nguyen et al., 2021 | Asia (Cambodia, Thailand and Vietnam) | Gekhos | Cloacal/fecal samples | 1318 | 293 | – |
| 188 | Pino-Vera et al., 2025 | Spain | Snake | Cloacal/fecal samples | 50 | 38 | 8 |
| 189 | Bruce et al., 2018 | UK | Tortoise | Cloacal/fecal samples | 89 | 5 | – |

**Supplementary Table S4.** The Joanna Briggs Institute (JBI) critical appraisal checklist for studies reporting prevalence data.

| Count | Authors & years | 1 | 2 | 3 | 4 | 5 | 6 | 7 | 8 | 9 | Total |
| --- | --- | --- | --- | --- | --- | --- | --- | --- | --- | --- | --- |
| 1 | Gopee, et al., 2000 | 1 | 1 | 1 | 1 | 1 | 1 | 1 | 1 | NA | 8 |
| 2 | Goupil et al., 2012 | 1 | 1 | 1 | 1 | 1 | 1 | 1 | 0 | NA | 7 |
| 3 | Fitzgerald et al., 2013 | 1 | 1 | 1 | 1 | 1 | 1 | 1 | 0 | NA | 7 |
| 4 | Jho et al., 2011 | 1 | 1 | 1 | 0 | 1 | 1 | 1 | 0 | NA | 6 |
| 5 | Pereira et al., 2017 | 1 | 1 | 1 | 1 | 1 | 1 | 1 | 0 | NA | 7 |
| 6 | Santoro et al., 2006 | 1 | 1 | 1 | 1 | 1 | 1 | 1 | 1 | NA | 8 |
| 7 | Plows et al., 1968 | 1 | 1 | 1 | 1 | 1 | 1 | 1 | 0 | NA | 7 |
| 8 | Schröter et al., 2006 | 1 | 1 | 1 | 1 | 1 | 1 | 1 | 0 | NA | 7 |
| 9 | Galgano et al., 2023 | 1 | 1 | 0 | 1 | 1 | 1 | 1 | 1 | NA | 7 |
| 10 | Centeno et al., 2020 | 1 | 1 | 1 | 1 | 1 | 1 | 1 | 1 | NA | 8 |
| 11 | Muñoz-Ibarra et al., 2022 | 1 | 1 | 1 | 1 | 1 | 1 | 1 | 1 | NA | 8 |
| 12 | Trotta et al., 2021 | 1 | 1 | 1 | 1 | 1 | 1 | 1 | 1 | NA | 8 |
| 13 | Guyomard-Rabenirina et al., 2020 | 1 | 1 | 1 | 1 | 1 | 1 | 1 | 1 | NA | 8 |
| 14 | Marin et al., 2022 | 1 | 1 | 1 | 1 | 1 | 1 | 1 | 1 | NA | 8 |
| 15 | Sharina et al., 2023 | 1 | 1 | 1 | 1 | 1 | 1 | 1 | 1 | NA | 8 |
| 16 | Hallinger et al., 2019 | 1 | 1 | 1 | 1 | 1 | 1 | 1 | 1 | NA | 8 |
| 17 | Bautista-Trujillo et al., 2020 | 1 | 1 | 1 | 1 | 1 | 1 | 1 | 1 | NA | 8 |
| 18 | Wheeler et al., 2012 | 1 | 1 | 1 | 1 | 1 | 1 | 1 | 1 | NA | 8 |
| 19 | Franco et al., 2011 | 1 | 1 | 1 | 1 | 1 | 1 | 1 | 1 | NA | 8 |
| 20 | Romero et al., 2015 | 1 | 1 | 1 | 1 | 1 | 1 | 1 | 1 | NA | 8 |
| 21 | Schmidt et al., 2014 | 1 | 1 | 1 | 1 | 1 | 1 | 1 | 1 | NA | 8 |
| 22 | Pawlak et al., 2020 | 1 | 1 | 1 | 1 | 1 | 1 | 1 | 0 | NA | 7 |
| 23 | Bemis et al., 2007 | 1 | 1 | 1 | 1 | 1 | 1 | 1 | 0 | NA | 7 |
| 24 | Kruse et al., 2015 | 1 | 1 | 0 | 1 | 1 | 1 | 1 | 1 | NA | 7 |
| 25 | Di Ianni et al., 2015 | 1 | 1 | 1 | 1 | 1 | 1 | 1 | 1 | NA | 8 |
| 26 | Ajayi et al., 2015 | 1 | 1 | 1 | 1 | 1 | 1 | 1 | 1 | NA | 8 |
| 27 | Song et al., 2023 | 1 | 1 | 1 | 1 | 1 | 1 | 1 | 1 | NA | 8 |
| 28 | Lecis et al., 2011 | 1 | 1 | 1 | 1 | 1 | 1 | 1 | 0 | NA | 7 |
| 29 | Cortes-Cortes et al., 2016 | 1 | 1 | 1 | 1 | 1 | 1 | 1 | 1 | NA | 8 |
| 30 | Heynol et al., 2015 | 1 | 1 | 1 | 1 | 1 | 1 | 1 | 1 | NA | 8 |
| 31 | Ives et al., 2017 | 1 | 1 | 1 | 1 | 1 | 1 | 1 | 1 | NA | 8 |
| 32 | da Silva Argôlo et al., 2013 | 1 | 1 | 1 | 1 | 1 | 1 | 1 | 0 | NA | 7 |
| 33 | Fasaei & Tamai, 2017 | 1 | 1 | 1 | 1 | 1 | 1 | 1 | 0 | NA | 7 |
| 34 | Marenzoni et al., 2022 | 1 | 1 | 1 | 1 | 1 | 1 | 1 | 1 | NA | 8 |
| 35 | Hacioglu & Tosunoglu, 2014 | 1 | 1 | 1 | 1 | 1 | 1 | 1 | 0 | NA | 7 |
| 36 | Somanath, 2022 | 1 | 1 | 1 | 1 | 1 | 1 | 1 | 0 | NA | 7 |
| 37 | Weerakhun et al., 2022 | 1 | 1 | 1 | 1 | 1 | 1 | 1 | 0 | NA | 7 |
| 38 | Ofori et al., 2023 | 1 | 1 | 1 | 1 | 1 | 1 | 1 | 1 | NA | 8 |
| 39 | Ogbodogbo et al., 2021 | 1 | 1 | 1 | 1 | 1 | 1 | 1 | 0 | NA | 7 |
| 40 | Wang et al., 2024 | 1 | 1 | 1 | 1 | 1 | 1 | 1 | 1 | NA | 8 |
| 41 | Junior et al., 2015 | 1 | 1 | 1 | 1 | 1 | 1 | 1 | 1 | NA | 8 |
| 42 | Lee et al., 2024 | 1 | 1 | 1 | 1 | 1 | 1 | 1 | 1 | NA | 8 |
| 43 | Morais et al., 2011 | 1 | 1 | 1 | 1 | 1 | 1 | 1 | 0 | NA | 7 |
| 44 | Mathewson, 1979 | 1 | 1 | 1 | 1 | 1 | 1 | 1 | 0 | NA | 7 |
| 45 | Chiacchio et al., 2014 | 1 | 1 | 1 | 1 | 1 | 1 | 1 | 1 | NA | 8 |
| 46 | Wang et al., 2020 | 1 | 1 | 1 | 1 | 1 | 1 | 1 | 1 | NA | 8 |
| 47 | Valença et al., 2025 | 1 | 1 | 1 | 1 | 1 | 1 | 1 | 1 | NA | 8 |
| 48 | Umbraško et al., 2023 | 1 | 1 | 1 | 1 | 1 | 1 | 1 | 1 | NA | 8 |
| 49 | Gruszynski et al., 2014 | 1 | 1 | 1 | 1 | 1 | 1 | 1 | 1 | NA | 8 |
| 50 | Ahasan et al., 2017 | 1 | 1 | 1 | 1 | 1 | 1 | 1 | 1 | NA | 8 |
| 51 | Eibach et al., 2019 | 1 | 1 | 1 | 1 | 1 | 1 | 1 | 0 | NA | 7 |
| 52 | Benhafid et al., 2025 | 1 | 1 | 1 | 1 | 1 | 1 | 1 | 1 | NA | 8 |
| 53 | Marin et al., 2013 | 1 | 1 | 1 | 1 | 1 | 1 | 1 | 1 | NA | 8 |
| 54 | Pace et al., 2019 | 1 | 1 | 1 | 1 | 1 | 1 | 1 | 1 | NA | 8 |
| 55 | Galosi et al., 2021 | 1 | 1 | 1 | 1 | 1 | 1 | 1 | 1 | NA | 8 |
| 56 | Maciel et al., 2010 | 1 | 1 | 1 | 1 | 1 | 1 | 1 | 1 | NA | 8 |
| 57 | Gong et al., 2014 | 1 | 1 | 1 | 1 | 1 | 1 | 1 | 1 | NA | 8 |
| 58 | Holgersson et al., 2016 | 1 | 1 | 1 | 1 | 1 | 1 | 1 | 1 | NA | 8 |
| 59 | Taiwo et al., 2021 | 1 | 1 | 1 | 1 | 1 | 1 | 1 | 1 | NA | 8 |
| 60 | Ramos et al., 2019 | 1 | 1 | 1 | 1 | 1 | 1 | 1 | 1 | NA | 8 |
| 61 | Adhikari et al., 2025 | 1 | 1 | 1 | 1 | 1 | 1 | 1 | 1 | NA | 8 |
| 62 | McWhorter et al., 2021 | 1 | 1 | 1 | 1 | 1 | 1 | 1 | 1 | NA | 8 |
| 63 | Nwachukwu et al., 2014 | 1 | 1 | 1 | 1 | 1 | 1 | 1 | 0 | NA | 7 |
| 64 | Bindaco et al., 2020 | 1 | 1 | 1 | 1 | 1 | 1 | 1 | 1 | NA | 8 |
| 65 | Abba et al., 2017 | 1 | 1 | 1 | 1 | 1 | 1 | 1 | 1 | NA | 8 |
| 66 | Oludairo, 2017 | 1 | 1 | 1 | 1 | 1 | 1 | 1 | 1 | NA | 8 |
| 67 | Burdick et al., 1984 | 1 | 1 | 1 | 1 | 1 | 1 | 1 | 0 | NA | 7 |
| 68 | Ríos et al., 2022 | 1 | 1 | 1 | 1 | 1 | 1 | 1 | 1 | NA | 8 |
| 69 | Uhart et al., 2011 | 1 | 1 | 1 | 1 | 1 | 1 | 1 | 1 | NA | 8 |
| 70 | Corrente et al., 2004 | 1 | 1 | 1 | 1 | 1 | 1 | 1 | 1 | NA | 8 |
| 71 | Kourany &Telford, 1981 | 1 | 1 | 1 | 1 | 1 | 1 | 1 | 1 | NA | 8 |
| 72 | Pace et al., 2019 | 1 | 1 | 1 | 1 | 1 | 1 | 1 | 1 | NA | 8 |
| 73 | Ruzhanova-Gospodinova et al., 2024 | 1 | 1 | 1 | 1 | 1 | 1 | 1 | 0 | NA | 7 |
| 74 | Marenzoni et al., 2015 | 1 | 1 | 1 | 1 | 1 | 1 | 1 | 1 | NA | 8 |
| 75 | Campagner et al., 2012 | 1 | 1 | 1 | 1 | 1 | 1 | 1 | 1 | NA | 8 |
| 76 | Wang et al., 2024 | 1 | 1 | 1 | 1 | 1 | 1 | 1 | 1 | NA | 8 |
| 77 | Molina-López et al., 2015 | 1 | 1 | 1 | 1 | 1 | 1 | 1 | 0 | NA | 7 |
| 78 | Bošnjak et al., 2016 | 1 | 1 | 1 | 1 | 1 | 1 | 1 | 1 | NA | 8 |
| 79 | Mengistu et al., 2022 | 1 | 1 | 1 | 1 | 1 | 1 | 1 | 1 | NA | 8 |
| 80 | Moreno et al., 1995 | 1 | 1 | 1 | 1 | 1 | 1 | 1 | 1 | NA | 8 |
| 81 | Mlangeni et al., 2024 | 1 | 1 | 1 | 1 | 1 | 1 | 1 | 1 | NA | 8 |
| 82 | Santana-Hernández et al., 2023 | 1 | 1 | 1 | 1 | 1 | 1 | 1 | 1 | NA | 8 |
| 83 | Ilayaraja et al., 2024 | 1 | 1 | 0 | 1 | 1 | 1 | 1 | 0 | NA | 6 |
| 84 | Yak et al., 2015 | 1 | 1 | 0 | 1 | 1 | 1 | 1 | 0 | NA | 6 |
| 85 | Pereira et al., 2017 | 1 | 1 | 1 | 1 | 1 | 1 | 1 | 1 | NA | 8 |
| 86 | Lazarkevich et al., 2024 | 1 | 1 | 1 | 1 | 1 | 1 | 1 | 1 | NA | 8 |
| 87 | Bertolini et al., 2021 | 1 | 1 | 0 | 1 | 1 | 1 | 1 | 0 | NA | 6 |
| 88 | Cristina et al., 2022 | 1 | 1 | 1 | 1 | 1 | 1 | 1 | 1 | NA | 8 |
| 89 | Pfleger et al., 2003 | 1 | 1 | 1 | 1 | 1 | 1 | 1 | 1 | NA | 8 |
| 90 | Dec et al., 2022 | 1 | 1 | 1 | 1 | 1 | 1 | 1 | 1 | NA | 8 |
| 91 | Schröter et al., 2004 | 1 | 1 | 1 | 1 | 1 | 1 | 1 | 1 | NA | 8 |
| 92 | Shnaiderman-Torban et al., 2019 | 1 | 1 | 1 | 1 | 1 | 1 | 1 | 1 | NA | 8 |
| 93 | Unger et al., 2024 | 1 | 1 | 1 | 0 | 1 | 1 | 1 | 0 | NA | 6 |
| 94 | Kurnianto et al., 2019 | 1 | 1 | 1 | 1 | 1 | 1 | 1 | 1 | NA | 8 |
| 95 | Jorge et al., 2023 | 1 | 1 | 1 | 1 | 1 | 1 | 1 | 0 | NA | 7 |
| 96 | Scheelings, 2008 | 1 | 1 | 1 | 1 | 1 | 1 | 1 | 1 | NA | 8 |
| 97 | Seepersadsingh & Adesiyun, 2003 | 1 | 1 | 1 | 1 | 1 | 1 | 1 | 0 | NA | 7 |
| 98 | Doden et al., 2021 | 1 | 1 | 1 | 1 | 1 | 1 | 1 | 1 | NA | 8 |
| 99 | Abatcha et al., 2013 | 1 | 1 | 1 | 1 | 1 | 1 | 1 | 1 | NA | 8 |
| 100 | Chen et al., 2010 | 1 | 1 | 1 | 1 | 1 | 1 | 1 | 1 | NA | 8 |
| 101 | Sumiyama et al., 2020 | 1 | 1 | 1 | 1 | 1 | 1 | 1 | 0 | NA | 7 |
| 102 | Tomastikova et al., 2017 | 1 | 1 | 1 | 1 | 1 | 1 | 1 | 1 | NA | 8 |
| 103 | Valley & Abatcha, 2014 | 1 | 1 | 1 | 0 | 1 | 1 | 1 | 1 | NA | 7 |
| 104 | Madsen, 1996 | 1 | 1 | 1 | 1 | 1 | 1 | 1 | 0 | NA | 7 |
| 105 | Liu et al., 2013 | 1 | 1 | 1 | 1 | 1 | 1 | 1 | 0 | NA | 7 |
| 106 | Scott, 1996 | 1 | 1 | 1 | 1 | 1 | 1 | 1 | 1 | NA | 8 |
| 107 | Burnham et al., 1998 | 1 | 1 | 1 | 1 | 1 | 1 | 1 | 1 | NA | 8 |
| 108 | Bastos et al., 2008 | 1 | 1 | 1 | 1 | 1 | 1 | 1 | 0 | NA | 7 |
| 109 | Adesiyun et al., 1998 | 1 | 1 | 1 | 1 | 1 | 1 | 1 | 1 | NA | 8 |
| 110 | Kuroki et al., 2019 | 1 | 1 | 1 | 1 | 1 | 1 | 1 | 0 | NA | 7 |
| 111 | Lukacet al., 2015 | 1 | 1 | 1 | 1 | 1 | 1 | 1 | 1 | NA | 8 |
| 112 | Sumiyama et al., 2020 | 1 | 1 | 1 | 1 | 1 | 1 | 1 | 1 | NA | 8 |
| 113 | Kuroki et al., 2013 | 1 | 1 | 1 | 1 | 1 | 1 | 1 | 1 | NA | 8 |
| 114 | Bjelland et al., 2020 | 1 | 1 | 1 | 1 | 1 | 1 | 1 | 1 | NA | 8 |
| 115 | Jegede et al., 2016 | 1 | 1 | 1 | 1 | 1 | 1 | 1 | 1 | NA | 8 |
| 116 | Nakadai et al., 2005 | 1 | 1 | 1 | 1 | 1 | 1 | 1 | 0 | NA | 7 |
| 117 | Back et al., 2016 | 1 | 1 | 1 | 1 | 1 | 1 | 1 | 0 | NA | 7 |
| 118 | Sharma et al., 2020 | 1 | 1 | 1 | 1 | 1 | 1 | 1 | 1 | NA | 8 |
| 119 | Baling & Mitchell, 2021 | 1 | 1 | 1 | 1 | 1 | 1 | 1 | 1 | NA | 8 |
| 120 | Kikillus et al., 2011 | 1 | 1 | 1 | 1 | 1 | 1 | 1 | 1 | NA | 8 |
| 121 | Moroni et al., 2025 | 1 | 1 | 1 | 1 | 1 | 1 | 1 | 1 | NA | 8 |
| 122 | Mairiga et al., 2024 | 1 | 1 | 1 | 1 | 1 | 1 | 1 | 0 | NA | 7 |
| 123 | Sylvester et al., 2014 | 1 | 1 | 1 | 1 | 1 | 1 | 1 | 0 | NA | 7 |
| 124 | Münch et al., 2012 | 1 | 1 | 1 | 0 | 1 | 1 | 1 | 0 | NA | 6 |
| 125 | De Silva et al., 2017 | 1 | 1 | 1 | 1 | 1 | 1 | 1 | 0 | NA | 7 |
| 126 | Madsen et al., 1998 | 1 | 1 | 1 | 1 | 1 | 1 | 1 | 1 | NA | 8 |
| 127 | Middleton et al., 2014 | 1 | 1 | 1 | 1 | 1 | 1 | 1 | 1 | NA | 8 |
| 128 | Guyomard-Rabenirina et al., 2019 | 1 | 1 | 1 | 1 | 1 | 1 | 1 | 1 | NA | 8 |
| 129 | Gopee et al., 2000 | 1 | 1 | 1 | 1 | 1 | 1 | 1 | 1 | NA | 8 |
| 130 | Corrente et al., 2017 | 1 | 1 | 1 | 1 | 1 | 1 | 1 | 1 | NA | 8 |
| 131 | Iveson et al., 1969 | 1 | 1 | 1 | 1 | 1 | 1 | 1 | 0 | NA | 7 |
| 132 | Shane et al., 1990 | 1 | 1 | 1 | 1 | 1 | 1 | 1 | 1 | NA | 8 |
| 133 | Abrahão et al., 2020 | 1 | 1 | 1 | 1 | 1 | 1 | 1 | 1 | NA | 8 |
| 134 | Geue & Löschner, 2002 | 1 | 1 | 1 | 1 | 1 | 1 | 1 | 1 | NA | 8 |
| 135 | Sánchez-Jiménez et al., 2011 | 1 | 1 | 1 | 1 | 1 | 1 | 1 | 1 | NA | 8 |
| 136 | Fieldsend et al., 2023 | 1 | 1 | 1 | 1 | 1 | 1 | 1 | 0 | NA | 7 |
| 137 | Dutton et al., 2013 | 1 | 1 | 1 | 1 | 1 | 1 | 1 | 0 | NA | 7 |
| 138 | Lockhart et al., 2008 | 1 | 1 | 1 | 1 | 1 | 1 | 1 | 0 | NA | 7 |
| 139 | Sá & Solari, 2001 | 1 | 1 | 1 | 1 | 1 | 1 | 1 | 0 | NA | 7 |
| 140 | Manolis et al., 1991 | 1 | 1 | 1 | 1 | 1 | 1 | 1 | 1 | NA | 8 |
| 141 | Zając et al., 2021 | 1 | 1 | 1 | 1 | 1 | 1 | 1 | 1 | NA | 8 |
| 142 | Hidalgo-Vila et al., 2007 | 1 | 1 | 1 | 1 | 1 | 1 | 1 | 0 | NA | 7 |
| 143 | Hidalgo-Vila et al., 2008 | 1 | 1 | 1 | 1 | 1 | 1 | 1 | 0 | NA | 7 |
| 144 | Clancy et al., 2016 | 1 | 1 | 1 | 1 | 1 | 1 | 1 | 1 | NA | 8 |
| 145 | Sumiyama et al., 2014 | 1 | 1 | 1 | 1 | 1 | 1 | 1 | 1 | NA | 8 |
| 146 | Bertelloni et al., 2016 | 1 | 1 | 1 | 1 | 1 | 1 | 1 | 0 | NA | 7 |
| 147 | Percipalle et al., 2011 | 1 | 1 | 1 | 1 | 1 | 1 | 1 | 0 | NA | 7 |
| 148 | Sting et al., 2013 | 1 | 1 | 1 | 0 | 1 | 1 | 1 | 1 | NA | 7 |
| 149 | Cummings et al., 2021 | 1 | 1 | 1 | 1 | 1 | 1 | 1 | 1 | NA | 8 |
| 150 | Wikström et al., 2014 | 1 | 1 | 1 | 1 | 1 | 1 | 1 | 0 | NA | 7 |
| 151 | Smith et al., 2012 | 1 | 1 | 1 | 1 | 1 | 1 | 1 | 1 | NA | 8 |
| 152 | Jiménez et al., 2015 | 1 | 1 | 1 | 1 | 1 | 1 | 1 | 1 | NA | 8 |
| 153 | Gay et al., 2014 | 1 | 1 | 1 | 1 | 1 | 1 | 1 | 1 | NA | 8 |
| 154 | Russo et al., 2018 | 1 | 1 | 1 | 1 | 1 | 1 | 1 | 0 | NA | 7 |
| 155 | Prapasarakul et al., 2012 | 1 | 1 | 1 | 1 | 1 | 1 | 1 | 1 | NA | 8 |
| 156 | Rush et al., 2020 | 1 | 1 | 1 | 1 | 1 | 1 | 1 | 0 | NA | 7 |
| 157 | Chambers & Hulse, 2006 | 1 | 1 | 1 | 1 | 1 | 1 | 1 | 1 | NA | 8 |
| 158 | Martínez et al., 2011 | 1 | 1 | 1 | 1 | 1 | 1 | 1 | 1 | NA | 8 |
| 159 | Scott & Foster, 1997. | 1 | 1 | 1 | 1 | 1 | 1 | 1 | 1 | NA | 8 |
| 160 | Lankau et al., 2012 | 1 | 1 | 1 | 1 | 1 | 1 | 1 | 1 | NA | 8 |
| 161 | Grupka et al., 2006 | 1 | 1 | 1 | 1 | 1 | 1 | 1 | 1 | NA | 8 |
| 162 | Callaway et al., 2011 | 1 | 1 | 1 | 1 | 1 | 1 | 1 | 1 | NA | 8 |
| 163 | Cambre et al., 1980 | 1 | 1 | 1 | 1 | 1 | 1 | 1 | 0 | NA | 7 |
| 164 | Bae et al., 2025 | 1 | 1 | 1 | 1 | 1 | 1 | 1 | 0 | NA | 7 |
| 165 | Merkevičienė et al., 2022 | 1 | 1 | 1 | 1 | 1 | 1 | 1 | 1 | NA | 8 |
| 166 | Zhao et al., 2024 | 1 | 1 | 1 | 1 | 1 | 1 | 1 | 1 | NA | 8 |
| 167 | Pedersen et al., 2009 | 1 | 1 | 1 | 1 | 1 | 1 | 1 | 0 | NA | 7 |
| 168 | Oros et al., 1998 | 1 | 1 | 0 | 1 | 1 | 1 | 1 | 0 | NA | 6 |
| 169 | Parsons et al., 2015 | 1 | 1 | 1 | 1 | 1 | 1 | 1 | 1 | NA | 8 |
| 170 | Prabhudev et al., 2018 | 1 | 1 | 1 | 1 | 1 | 1 | 1 | 0 | NA | 7 |
| 171 | Dipineto et al., 2012 | 1 | 1 | 1 | 1 | 1 | 1 | 1 | 1 | NA | 8 |
| 172 | Dégi et al., 2023 | 1 | 1 | 1 | 1 | 1 | 1 | 1 | 1 | NA | 8 |
| 173 | Gordon & Cowling, 2003 | 1 | 1 | 1 | 1 | 1 | 1 | 1 | 1 | NA | 8 |
| 174 | Pulford et al., 2019 | 1 | 1 | 1 | 1 | 1 | 1 | 1 | 1 | NA | 8 |
| 175 | Dehghani et al., 2016 | 1 | 1 | 1 | 1 | 1 | 1 | 1 | 0 | NA | 7 |
| 176 | Nguyen et al., 2018 | 1 | 1 | 1 | 1 | 1 | 1 | 1 | 1 | NA | 8 |
| 177 | Otis & Behler, 1973 | 1 | 1 | 1 | 1 | 1 | 1 | 1 | 0 | NA | 7 |
| 178 | Książczyk et al., 2021 | 1 | 1 | 1 | 1 | 1 | 1 | 1 | 0 | NA | 7 |
| 179 | Romero et al., 2016 | 1 | 1 | 1 | 1 | 1 | 1 | 1 | 0 | NA | 7 |
| 180 | Middleton, 2008 | 1 | 1 | 1 | 1 | 1 | 1 | 1 | 1 | NA | 8 |
| 181 | Rose, 2021 | 1 | 1 | 1 | 1 | 1 | 1 | 1 | 1 | NA | 8 |
| 182 | Thaller et al., 2010 | 1 | 1 | 1 | 1 | 1 | 1 | 1 | 0 | NA | 7 |
| 183 | Mlangeni et al., 2025 | 1 | 1 | 1 | 1 | 1 | 1 | 1 | 0 | NA | 7 |
| 184 | Casey, 2011 | 1 | 1 | 1 | 1 | 1 | 1 | 1 | 1 | NA | 8 |
| 185 | Pawlak et al., 2024 | 1 | 1 | 1 | 1 | 1 | 1 | 1 | 0 | NA | 7 |
| 186 | Dec et al., 2022 | 1 | 1 | 1 | 1 | 1 | 1 | 1 | 1 | NA | 8 |
| 187 | Nguyen et al., 2021 | 1 | 1 | 1 | 1 | 1 | 1 | 1 | 1 | NA | 8 |
| 188 | Pino-Vera et al., 2025 | 1 | 1 | 1 | 1 | 1 | 1 | 1 | 1 | NA | 8 |
| 189 | Bruce et al., 2018 | 1 | 1 | 1 | 1 | 1 | 1 | 1 | 0 | NA | 7 |

(1) Was the sample frame appropriate to address the target population? (2) Were study participants recruited in an appropriate way? (3) Was the sample size adequate? (4) Were the study subjects and setting described in detail? (5) Was the data analysis conducted with sufficient coverage of the identified sample? (6) Were valid methods used for the identification of the condition? (7) Was the condition measured in a standard, reliable way for all participants? (8) Was there appropriate statistical analysis? (9) Was the response rate adequate, and, if not, was the low response rate managed appropriately?

**Supplementary Table S5.** Subgroup analysis of *Salmonella* spp. and *E. coli* in reptiles reported worldwide stratified by countries.

| **Countries** | **Number of studies** | **Pooled estimates** | | | **Measure of heterogeneity** | | | |
| --- | --- | --- | --- | --- | --- | --- | --- | --- |
|  |  | **Sample**  **size** | **Positive Isolates** | ***I^2^* (95%CI)** | **Q-Value** | ***I^2^*** | ***df* [Q]** | ***P*-Value** |
| **South Africa** | | | | | | | | |
| *Salmonella* spp. | 2 | 29 | 39 | 34.7 (03.5-88.6) | 6.220 | 83.924 | 1 | – |
| *E. coli* | 2 | 46 | 24 | 79.2 (29.5-97.2) | 2.557 | 60.890 | 1 | – |
| **Spain** | | | | | | | | |
| *Salmonella* spp. | 12 | 1059 | 273 | 22.9 (13.8-35.6) | 151.134 | 92.722 | 11 | 0.410 |
| *E. coli* | 2 | 141 | 25 | 17.8 (12.3-25.0) | 0.159 | 0.000 | 1 | – |
| **USA** | | | | | | | | |
| *Salmonella* spp. | 26 | 2185 | 791 | 39.8 (27.6-53.4) | 476.944 | 94.758 | 25 | 0.332 |
| *E. coli* | 4 | 197 | 169 | 28.6 (10.5-57.9) | 20.561 | 85.409 | 3 | 0.496 |
| **Indonesia** | | | | | | | | |
| *Salmonella* spp. | 2 | 315 | 311 | 98.7 (88.1-99.9) | 2.716 | 63.187 | 1 | – |
| *E. coli* | 2 | 315 | 330 | 98.7 (88.1-99.9) | 2.716 | 63.187 | 1 | – |
| **Italy** | | | | | | | | |
| *Salmonella* spp. | 13 | 1327 | 384 | 27.5 (19.5-37.2) | 122.396 | 90.196 | 12 | 0.131 |
| *E. coli* | 2 | 128 | 6 | 04.8 (02.2-10.3) | 0.337 | 0.000 | 1 | – |
| **Malaysia** | | | | | | | | |
| *Salmonella* spp. | 4 | 151 | 36 | 19.8 (07.9-41.7) | 15.880 | 81.109 | 3 | 0.308 |
| *E. coli* | 2 | 67 | 7 | 10.5 (05.1-20.4) | 0.021 | 0.000 | 1 | – |
| **Nigeria** | | | | | | | | |
| *Salmonella* spp. | 3 | 972 | 261 | 16.7 (09.8-26.8) | 10.205 | 80.402 | 2 | 0.601 |
| *E. coli* | 6 | 695 | 261 | 37.1 (18.9-59.9) | 100.146 | 95.007 | 5 | 0.188 |
| **Poland** | | | | | | | | |
| *Salmonella* spp. | 4 | 877 | 685 | 58.0 (26.3-84.3) | 114.237 | 97.374 | 3 | 0.174 |
| *E. coli* | 2 | 187 | 56 | 32.3 (11.8-62.9) | 15.025 | 93.344 | 1 | – |
| **Australia** | | | | | | | | |
| *Salmonella* spp. | 7 | 1542 | 767 | 43.8 (24.7-65.0) | 223.354 | 97.314 | 6 | 0.652 |
| *E. coli* | 3 | 459 | 238 | 57.4 (15.4-90.9) | 64.987 | 96.922 | 2 | 0.601 |
| **Brazil** | | | | | | | | |
| *Salmonella* spp. | 12 | 856 | 287 | 31.3 (17.6-49.5) | 161.361 | 93.183 | 11 | 0.217 |
| *E. coli* | 8 | 500 | 108 | 21.8 (09.5-42.5) | 97.655 | 92.832 | 7 | 0.216 |
| **New Zealand** | | | | | | | | |
| *Salmonella* spp. | 5 | 2373 | 110 | 04.4 (02.2-08.6) | 47.093 | 91.506 | 4 | 0.327 |
| **Thailand** | | | | | | | | |
| *Salmonella* spp. | 2 | 385 | 221 | 91.1 (02.5-100.0) | 18.477 | 94.588 | 1 | – |
| **Iran** | | | | | | | | |
| *Salmonella* spp. | 2 | 28 | 12 | 38.3 (09.2-79.2) | 4.054 | 75.339 | 1 | – |
| **Korea** | | | | | | | | |
| *Salmonella* spp. | 3 | 89 | 40 | 40.3 (18.1-67.2) | 10.055 | 80.109 | 2 | 0.601 |
| **Japan** | | | | | | | | |
| *Salmonella* spp. | 6 | 1355 | 408 | 40.3 (14.1-73.6) | 350.367 | 98.573 | 5 | 0.090 |
| **China** | | | | | | | | |
| *Salmonella* spp. | 5 | 1037 | 235 | 32.4 (12.6-61.5) | 192.015 | 97.917 | 4 | 0.141 |
| **Trinidad** | | | | | | | | |
| *Salmonella* spp. | 2 | 187 | 27 | 14.4 (10.1-20.2) | 0.000 | 0.000 | 1 | – |
| **Ecuador** | | | | | | | | |
| *E. coli* | 2 | 114 | 157 | 86.8 (33.4-98.8) | 3.525 | 71.630 | 1 | – |
| **Romania** | | | | | | | | |
| *Salmonella* spp. | 2 | 456 | 47 | 16.1 (01.3-74.1) | 63.780 | 98.432 | 1 | – |
| **Czech Republic** | | | | | | | | |
| *Salmonella* spp. | 2 | 231 | 18 | 15.8 (01.1-76.5) | 26.629 | 96.245 | 1 | – |
| **Bulgaria** | | | | | | | | |
| *E. coli* | 2 | 139 | 5 | 03.6 (01.5-08.4) | 0.002 | 0.000 | 1 | – |
| **Germany** | | | | | | | | |
| *Salmonella* spp. | 7 | 1583 | 555 | 41.4 (32.7-50.7) | 41.362 | 85.494 | 6 | 0.050 |
| **Mexico** | | | | | | | | |
| *E. coli* | 3 | 358 | 260 | 64.1 (19.0-93.2) | 47.647 | 95.802 | 2 | 0.601 |
| **Caribbean** | | | | | | | | |
| *Salmonella* spp. | 3 | 197 | 52 | 24.5 (06.3-60.9) | 33.031 | 93.945 | 2 | 0.601 |
| **Ghana** | | | | | | | | |
| *Salmonella* spp. |  |  |  |  |  |  |  | – |
| *E. coli* | 2 | 67 | 36 | 50.0 (02.5-97.5) | 26.743 | 96.261 | 1 | – |
| **Zimbabwe** | | | | | | | | |
| *Salmonella* spp. | 2 | 227 | 52 | 23.0 (18.0-28.9) | 0.840 | 0.000 | 1 | – |

**Supplementary Table S6.** Subgroup analysis of *Salmonella* spp. and *E. coli* in reptiles reported worldwide stratified by samples and detection methods.

| **Sample type and Detection methods** | **Number of studies** | **Sample**  **size** | **Positive Isolates** | ***I^2^* (95%CI)** | **Q-Value** | ***I^2^*** | ***df* [Q]** | ***P*-Value** |
| --- | --- | --- | --- | --- | --- | --- | --- | --- |
| **Faecal samples** | | | | | | | | |
| *Salmonella* spp*.* | 10 | 1896 | 721 | 45.8 (32.5-59.8) | 113.931 | 92.100 | 9 | 0.179 |
| *E. coli* | 6 | 821 | 280 | 40.5 (07.6-84.9) | 189.015 | 97.355 | 5 | 0.850 |
| **Oral + cloacal swabs** | | | | | | | | |
| *Salmonella* spp. | 6 | 475 | 76 | 13.4 (07.2-23.6) | 21.272 | 76.495 | 5 | 0.573 |
| *E. coli* | 6 | 305 | 75 | 24.9 (13.5-41.4) | 22.132 | 77.408 | 5 | 0.850 |
| **Cloacal samples** | | | | | | | | |
| *Salmonella* spp*.* | 15 | 919 | 187 | 21.9 (15.9-29.4) | 67.557 | 79.277 | 14 | 0.766 |
| *E. coli* | 7 | 618 | 215 | 21.1 (07.7-45.9) | 132.316 | 95.465 | 6 | 0.010 |
| **Cloacal/faecal samples** | | | | | | | | |
| *Salmonella* spp. | 108 | 16577 | 5277 | 35.1 (30.5-40.1) | 2795.921 | 96.173 | 107 | 0.754 |
| *E. coli* | 27 | 2577 | 1125 | 31.8 (22.3-43.2) | 494.934 | 94.747 | 26 | 0.818 |
| **Oral swabs** | | | | | | | | |
| *Salmonella* spp*.* | 6 | 135 | 24 | 14.9 (03.2-48.2) | 34.703 | 85.592 | 5 | 0.573 |
| *E. coli* | 5 | 115 | 20 | 18.0 (10.9-28.3) | 4.842 | 17.392 | 4 | 0.624 |
| **Other Samples** | | | | | | | | |
| *Salmonella* spp*.* | 11 | 1864 | 209 | 23.5 (11.6-41.8) | 223.5516 | 95.526 | 10 | 0.585 |
| *E. coli* | 6 | 557 | 78 | 20.3 (05.3-53.9) | 108.406 | 95.388 | 5 | 0.573 |
| **Culture + MALDI-TOF MS** | | | | | | | | |
| *Salmonella* spp. | 2 | 208 | 22 | 08.9 (02.1-30.1) | 7.116 | 85.947 | 1 | – |
| *E. coli* | 2 | 208 | 8 | 03.9 (01.9-07.5) | 0.028 | 0.00 | 1 | – |
| **WGS** | | | | | | | | |
| *Salmonella* spp. | 2 | 664 | 115 | 20.1 (04.4-58.0) | 63.629 | 98.428 | 1 | – |
| **Culture + serotyping + PCR** | | | | | | | | |
| *Salmonella* spp*.* | 6 | 656 | 199 | 32.8 (13.7-59.9) | 117.816 | 95.756 | 5 | 0.850 |
| **Culture + PCR** | | | | | | | | |
| *Salmonella* spp. | 12 | 1471 | 241 | 22.1 (13.0-35.0) | 178.427 | 93.835 | 11 | 0.583 |
| *E. coli* | 10 | 1144 | 147 | 14.9 (06.0-32.5) | 194.705 | 95.378 | 9 | 0.531 |
| **Culture + serotyping** | | | | | | | | |
| *Salmonella* spp*.* | 9 | 680 | 325 | 48.1 (35.6-60.9) | 68.445 | 88.312 | 8 | 0.754 |
| **Culture + Biochemical** | | | | | | | | |
| *Salmonella* spp. | 27 | 2327 | 610 | 26.9 (18.8-37.0) | 387.999 | 93.299 | 26 | 0.770 |
| *E. coli* | 31 | 2236 | 695 | 23.1 (16.6-31.2) | 314.513 | 90.461 | 30 | 0.825 |
| **Culture + Biochemical + serotyping** | | | | | | | | |
| *Salmonella* spp. | 90 | 15293 | 5071 | 35.5 (29.9-41.4) | 2874.526 | 96.904 | 89 | 0.329 |
| *E. coli* | 6 | 738 | 451 | 50.3 (32.2-68.3) | 59.284 | 91.566 | 5 | 0.347 |

**A.** *E. coli* in cloacal samples

**B.** *Salmonella* spp. studies conducted in Africa

**Supplementary Figure S1.** Funnel plot showing the evidence of publication bias of the studies investigating *E. coli* in cloacal samples (**A**) and studies investigating *Salmonella* spp. conducted in Africa (**B**).
